# Can mental health diagnoses in administrative data be used for research? A systematic review of the accuracy of routinely collected diagnoses

**DOI:** 10.1186/s12888-016-0963-x

**Published:** 2016-07-26

**Authors:** Katrina A. S. Davis, Cathie L. M. Sudlow, Matthew Hotopf

**Affiliations:** 1 Department of Psychological Medicine, Institute of Psychiatry Psychology and Neuroscience, Kings College London, London, UK; 2 South London and Maudsley NHS Foundation Trust, Maudsley Hospital, Denmark Hill, London, SE5 8AZ UK; 3 Department of Psychological Medicine and SLaM/IoPPN BRC, Kings College London, PO62, Weston Education Centre, Cutcombe Road, London, SE5 9RJ UK

**Keywords:** Psychiatry, Diagnosis, Population research, Administrative data, Electronic health records, Case registers, Hospital episode statistics

## Abstract

**Background:**

There is increasing availability of data derived from diagnoses made routinely in mental health care, and interest in using these for research. Such data will be subject to both diagnostic (clinical) error and administrative error, and so it is necessary to evaluate its accuracy against a reference-standard. Our aim was to review studies where this had been done to guide the use of other available data.

**Methods:**

We searched PubMed and EMBASE for studies comparing routinely collected mental health diagnosis data to a reference standard. We produced diagnostic category-specific positive predictive values (PPV) and Cohen’s kappa for each study.

**Results:**

We found 39 eligible studies. Studies were heterogeneous in design, with a wide range of outcomes. Administrative error was small compared to diagnostic error. PPV was related to base rate of the respective condition, with overall median of 76 %. Kappa results on average showed a moderate agreement between source data and reference standard for most diagnostic categories (median kappa = 0.45–0.55); anxiety disorders and schizoaffective disorder showed poorer agreement. There was no significant benefit in accuracy for diagnoses made in inpatients.

**Conclusions:**

The current evidence partly answered our questions. There was wide variation in the quality of source data, with a risk of publication bias. For some diagnoses, especially psychotic categories, administrative data were generally predictive of true diagnosis. For others, such as anxiety disorders, the data were less satisfactory. We discuss the implications of our findings, and the need for researchers to validate routine diagnostic data.

**Electronic supplementary material:**

The online version of this article (doi:10.1186/s12888-016-0963-x) contains supplementary material, which is available to authorized users.

## Background

Databases such as those produced by electronic health records or for reimbursement of medical costs, contain routinely collected data on diagnosis that has considerable application in research, such as for ascertaining outcomes in epidemiology or identifying suitable research participants for clinical trials [1–3]. There has been a long history of using routine data in mental health research, from the earliest studies of asylum records through to the ‘case register’ of the 20th century [4]. The easy availability of large volumes of data regarding patients with mental health diagnoses from routine clinical practice following the shift to electronic health records can be utilised for research [5, 6], and massed electronically produced administrative data has been used by a diverse range of groups, using routinely collected diagnosis to identify cases of mental illness for public health and advocacy [7–10].

Biobanks may also link to administrative databases: connecting genomic, physiological and self-report data with hospital episodes and death registration, to become powerful tools to gain insight into risk and protective factors of a wide range of diseases. UK Biobank recruited 500,000 people aged between 40 and 69 years in 2006–2010 from across the UK [11], and data linkage includes to Hospital Episode Statistics (HES) in England, and the equivalent datasets in Scotland and Wales, which log every hospital admission, including to psychiatric hospitals, and include ICD-10 diagnosis codes (WHO’s International Classification of Diseases) [12]. Such linkages provide a means of greatly enriching UK Biobank’s outcomes in a cost-effective and scalable manner, and there would be the opportunity for identifying cases of psychiatric illness through ICD-10 codes from HES and other records. Similar data linkages are in place for other large studies [4].

Despite the promise of data linkage, there are inevitably concerns that routine data, collected for non-research purposes, may be prone to misclassification. Accuracy can be affected by errors at a number of points, broadly described as “diagnostic error” and “administrative error”. Diagnostic error occurs when the clinician fails to find the signs/symptoms of the correct condition, makes a diagnosis not supported by research criteria, or records a diagnosis at odds with their real conclusion. Administrative error involves issues around turning the physician diagnosis into codes (ICD in the case of Hospital Episode Statistics), and submitting these codes attached to the correct record and identifiers. “Coding” traditionally utilised trained non-clinical administrators interpreting the treating clinician’s handwritten records to derive a valid ICD code for the record [13], which is inevitably error-prone – although in the age of electronic health records, where the clinicians generally assign diagnosis codes, data entry error and miscoding still occurs [5, 14, 15].

Recent reviews of accuracy of English HES data have mainly concentrated on administrative error [1, 16, 17], and there is a lack of specific information on diagnostic accuracy for psychiatric disorders. In mental health there may be particular issues about diagnostic error, which would be reflected in evaluations of the quality of psychiatric diagnoses in other data sources [15, 18]. This may help when considering using HES and other such administrative databases to identify cases of mental illness. A previous attempt to collate results from a variety of psychiatric databases by Byrne et al. from Kings College London in 2005, identified that papers were mostly of poor quality, and the results were too variable to give an overall view on diagnostic validity [19].

The aim of the present systematic review was to identify and collate results regarding the accuracy of diagnosis in routinely collected data from mental health settings to guide the interpretation of the use of such data to identify cases. Specifically our objectives were: to evaluate the agreement and validity of a routinely recorded diagnosis compared with a reference diagnosis for psychiatric disorders (i) in general, (ii) for different psychiatric diagnoses, and (iii) comparing diagnoses made as inpatients with outpatients.

## Methods

We used Preferred Reporting Items for Systematic Reviews and Meta-Analyses (PRISMA) guidelines to develop the design, conduct and reporting of this review. One author (KD) carried out the search and extracted data.

### Search strategy

We searched Medline (PubMed) and Embase from 1980 to November 2014 for studies assessing the accuracy of routinely collected data regarding psychiatric diagnosis against a reference standard diagnosis. We used a combination of medical subject heading and text word terms for ‘mental health’; ‘accuracy’, ‘reliability’ and ‘validity’; ‘diagnosis’, ‘ICD’ and ‘DSM’; and ‘medical records’, ‘coding’ or ‘registers’. We reviewed bibliographies of included publications and used Google Scholar to identify any citing papers for additional relevant reviews or studies (see Additional files 1 and 2: Figure S1 and S2 for detail of search strategy).

### Eligibility criteria

Studies were included if they were a peer-reviewed published comparison of psychiatric diagnoses in routinely recorded data against reference standard diagnoses using ICD, DSM or similar psychiatric classification systems. The studies included samples of patients recruited from population, primary or secondary care settings; however, the diagnoses under study were those derived from secondary care only - either inpatient or outpatient psychiatric services. The data that was being examined (source diagnosis) could be taken from official clinical documentation [“clinical”] or from a research or administrative database. Where a clinical source diagnosis was used, the comparison data (reference diagnosis) had to be a research diagnosis [“research”] to look at *diagnostic error*, but where a database source diagnosis was used, clinical documentation [“chart”] could also be used for a reference diagnosis to look at *administrative error*. Comparing a database source diagnosis and a research reference diagnosis gives clinical and administrative error combined. Research diagnoses could be considered reference diagnoses whether they used structured casenote review and/or research interview to reach the diagnosis, as long as they conformed to Spitzer’s “Longitudinal, Expert and All Data” (LEAD) diagnostic approach [20]. Studies were reviewed for inclusion by KD, and where there was doubt, discussed with MH.

We assessed each eligible paper for quality using an established checklist [21] which marks studies on aims (3 marks), method (9), results & discussion (10). There were no suggested cut-off points with this checklist, so we defined criteria for inadequate, poor, moderate and good quality using total and category-specific scores. The studies considered inadequate were those which scored less than two in any category or less than ten overall. Studies were considered good quality if they scored at least 75 % of the points from each section (see Additional file 3: Table S1 for the quality rating of individual papers).

### Data extraction

We devised a form to extract information from each study which included (1) the nature of the cohort studied, including clinical setting, selection criteria, location, sample size and age range; (2) source of routine diagnostic data; (3) nature of reference diagnosis, how it was derived, and any measures of reliability for this diagnosis; (4) the diagnosis, diagnostic grouping or diagnoses under study, and the diagnostic system used (e.g. ICD/DSM); (5) the base rate for each diagnosis studied (i.e. the prevalence in the setting the diagnosis was made according to reference diagnosis); (6) measures of concordance between diagnostic data and reference diagnosis: validity measures – sensitivity, specificity, positive- and negative-predictive values – and agreement measures – percentage agreement, Cohen’s kappa (k) and area under the curve.

### Data analysis

After consideration of the data available from the papers, and our aim to assess the accuracy of case finding by using routine diagnosis we chose two parameters to report: (1) Positive predictive value (PPV) provides an estimate of the probability that a given diagnosis in the source data will match the reference diagnosis acting as “gold standard”; (2) Cohen’s kappa provides a measure of agreement between the source data and the reference comparison. The sensitivity and negative predictive value are useful for considering representativeness and the recruitment of controls, but they are of most use when using true population studies, where unidentified cases can be found, rather than the secondary care studies identified here.

We give diagnosis-specific results at chapter level (eg “affective disorders”) and disorder level (eg “bipolar affective disorder”) according to the reporting in the original papers. Some papers report at both chapter level and disorder level, in which case the results for the disorder will be a subset of the results for the chapter. Otherwise, we treated results within the same study as independent for data analysis purposes.

Using cross-tabulations provided in the source paper, or working back from accuracy statistics, a 2x2 table was constructed of true-positives, false-positives, false-negatives, and true negatives for each diagnosis studied in each paper. From this, the PPV and percentage agreement was calculated. It was thus possible to calculate a PPV for all of the specific outcome categories, even where not originally reported, with 95 % confidence intervals calculated using Wilson’s method [22].

Cohen’s kappa was calculated from the observed and expected agreement [23]. Two difficulties were encountered: (i) where no-one without the diagnosis in source data was studied, kappa could not be calculated; (ii) where agreement was worse than chance, a negative kappa results; since the magnitude of a negative kappa is uninformative this was regarded as zero.

We did not undertake formal meta-analysis or meta-regression due to the heterogeneity between studies in their methods, participant characteristics and reporting. We used non-parametric tests – Kruskall-Wallis H with Bonferroni correction - to assess for independence of groups for data source, and to explore setting of diagnosis. Calculations and graphs were performed using Microsoft Excel 2013 with the Real Statistics plug-in [24].

## Results

### Papers

**Fig. 1:**
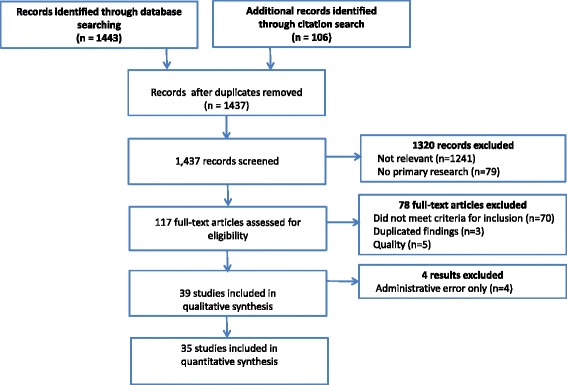
Preferred Reporting Items for Systematic Reviews and Meta-Analyses (PRISMA) flow diagram of the systematic review

Figure 1 shows the PRIMSA flow chart for the review. Our literature search identified 117 potential publications. Of these 72 were excluded, and a further six were found to be of inadequate quality, leaving a total of 39 [25–63]. The excluded papers and reasons for exclusion are in Additional file 4: Table S2.

Included studies are described in Additional file 5: Table S3. The publications were predominantly Scandinavian (*n* = 22) and from the USA (*n* = 10), with the four largest studies coming from Canada. They were published between 1988 and 2014 although they reflect diagnoses made up to 20 years prior to the date of publication of the studies. Many had been published with a view to using the source data for further research.

### Study design

Cohorts ranged from samples of the general population to inpatients with specified working diagnosis. The prevalence of specified diagnoses in secondary care (base rates) varied widely. The number of diagnostic categories examined in each study varied between one and eight. In all, there were 16 diagnostic categories considered. In the 39 papers studied, there were 104 diagnosis-specific results. The most common diagnosis studied was schizophrenia (*n* = 19), followed by bipolar affective disorder (*n* = 12) and unipolar depression (*n* = 12). Ten results showed the overall agreement across a number of diagnoses. A number of studies used the category of “schizophrenia spectrum” (*n* = 13) to describe a group of psychotic disorders – usually including schizophrenia, schizotypal disorder and schizoaffective disorder, but varying on the inclusion of other schizophreniform psychoses and delusional disorders. Since the studies were comparing like-for-like in their routine and reference diagnoses, we used the term “schizophrenia spectrum” whenever a group of non-affective psychoses including schizophrenia was studied, without further differentiation.

The source data was derived directly from clinical notes in 13 studies (57 diagnosis-specific results), while 26 studies used databases: 17 used regional and national research databases; nine larger studies used databases created primarily for administrative purposes. The reference diagnosis was the “chart” diagnosis in four studies, and was otherwise a research diagnosis. Research diagnoses consisted of a notes review in 15, an interview in five, and an interview with notes review in 15. Thirteen studies used more than one researcher reaching a diagnosis independently and reported the inter-rater reliability of the research diagnosis. In 11 cases, this could be compared with the kappa agreement between source and reference [33, 34, 36, 38, 44–46, 50, 57, 59, 61].

There are three groups of results: those using a database diagnosis as the source and chart diagnosis as the reference, giving *administrative error only* (six results from four papers); those using clinical diagnosis as the source with research diagnosis as the reference, giving *diagnostic error only* (57 results from 13 papers); and those using database diagnosis as the source with research diagnosis as the reference, giving *administrative and diagnostic error combined* (41 results from 22 papers).

Twenty-four studies examined diagnoses made as an inpatient, while 13 included diagnoses recorded as in- or out-patients; with two exclusively examining data from outpatients. Eight studies concentrated on diagnoses made at first presentation. Two studies [40, 55] specified that more than one entry stating the diagnosis was required for inclusion in the cohort, and a further two [25, 43] selected inpatients with one diagnosis, but outpatients only if they had two. Multiple instances of diagnosis were the norm in the remainder of the studies, except those of first episode, with various algorithms for treating differing diagnoses: “at least one”, “last”, “most often” and using a formal hierarchy. The result using the “last” diagnosis was chosen for this analysis where multiple results were given, as this was shown to be a good method [54] and thought to be most similar to where no choice in results had been given.

The source data were coded using systems from DSM versions III, III-R & IV or ICD versions 7–10 or local codes based on these classifications (eg a Canadian version of ICD-10 or codes specific to Veterans Affairs). Frequently the administrative diagnoses covered a long time frame, and therefore mixtures of editions were used. For example McConville collected data from 1962 to 1996, covering ICD versions 7, 8, 9 and 10 [45].

### Outcomes

There was a wide range of PPVs, from 10 to 100 % with an overall median of 76 % and a negative skew. PPV is connected mathematically to the base rate of the condition, and simple linear regression confirmed a moderate positive association between PPV and prevalence (*r*^2^ = 0.27, *p* < <0.01, correlation coefficient (β) =0.40,). Kappa was calculated for the 29 studies where a “true negative” rate was known, giving 91 diagnosis-specific results. Agreement using kappa ranged from <0 to 1 (i.e., from worse than chance agreement to a perfect match), and the distribution was fairly symmetrical. The median kappa was 0.49, a value that is classed as a moderate inter-rater agreement [64]. In contrast with PPV, there was no correlation with prevalence (*r*^2^ = 0.0032, *p* = 0.97). Due to the dependence of PPV on prevalence, kappa would be the preferred statistic when comparing between data sources with different prevalence. The kappa values can also be compared against the inter-rater reliability of the research diagnoses in eleven of the papers. In all cases the kappa result shows greater discordance for the source data than between researchers: kappa for research diagnosis was 0.71–1, being between 1.2 to 3 times higher (median 1.7) than the results for source data. This suggests that the studies are demonstrating more than the reliability of the diagnostic codes.

The median PPV and kappa results for the administrative error group were 91 % and 0.73 respectively; for the diagnostic error group 74 % and 0.48; for the combined error group 77 % and 0.36. Kruskal Wallis pairwise testing confirmed that kappa was higher for the administrative error group versus the combined group (*p* = 0.006), and not significantly different for the diagnostic error versus the combined groups (*p* = 0.33). The significantly higher kappa agreement for the administrative error only group suggests that the error in diagnostic data overall occurs mainly at the clinical rather than the administrative stage. A few papers were able to comment directly on the relative contribution of clinical versus administrative error. Moilenan et al. [46] and Makikyro et al. [44] agreed, with clinical errors greatly outnumbering administrative ones (55 vs 9 and 16 vs 2 respectively); although Uggerby et al. [60] was at odds, with seven clinical vs 13 administrative errors in their research database.

We omitted the administrative error only group from further analysis, and the results from the diagnostic error only group and the combined error group were considered together for subsequent comparisons.

### Results by diagnostic group

**Fig. 2:**
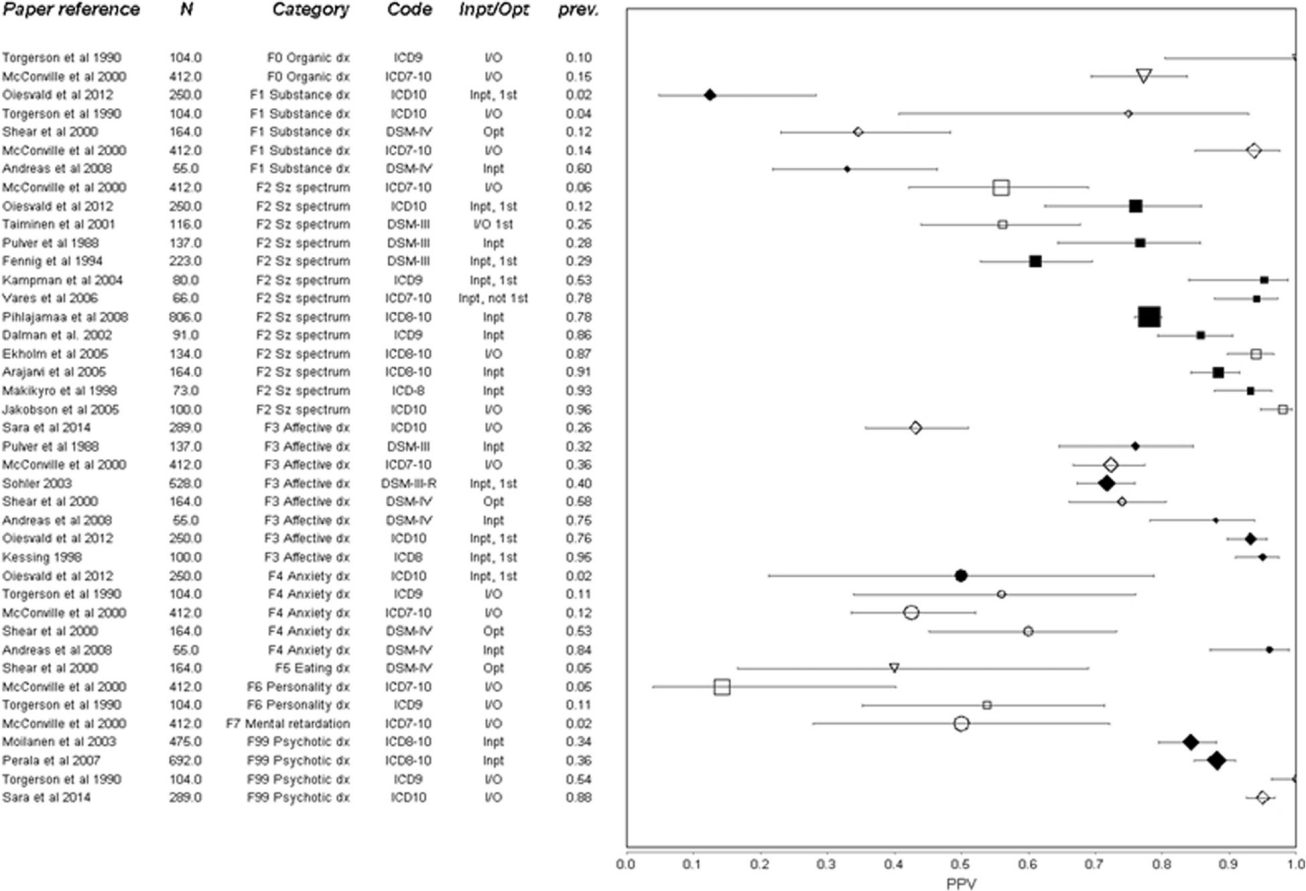
Forest Plot of positive predictive value (PPV) of routine diagnoses for the respective research diagnosis at a chapter level chapter-level. Whiskers represent 95 % confidence intervals. Sorted by diagnosis (also indicated by *shape*), then prevalence in the sample *prev.* Diagnosis has been converted to nearest ICD-10 equivalent, with original code indicated. *Size* of marker represents square root of complete sample size N. Inpatient (Inpt), Outpatient (Opt) or mixed (I/O) origin of data indicated, with studies using only inpatient diagnoses also having *filled* marker. Studies using only people in their first episode of illness (1st) also identified. Abbreviations: Dx = disorder/disorders Sz spectrum = schizophrenia spectrum disorders

**Fig. 3:**
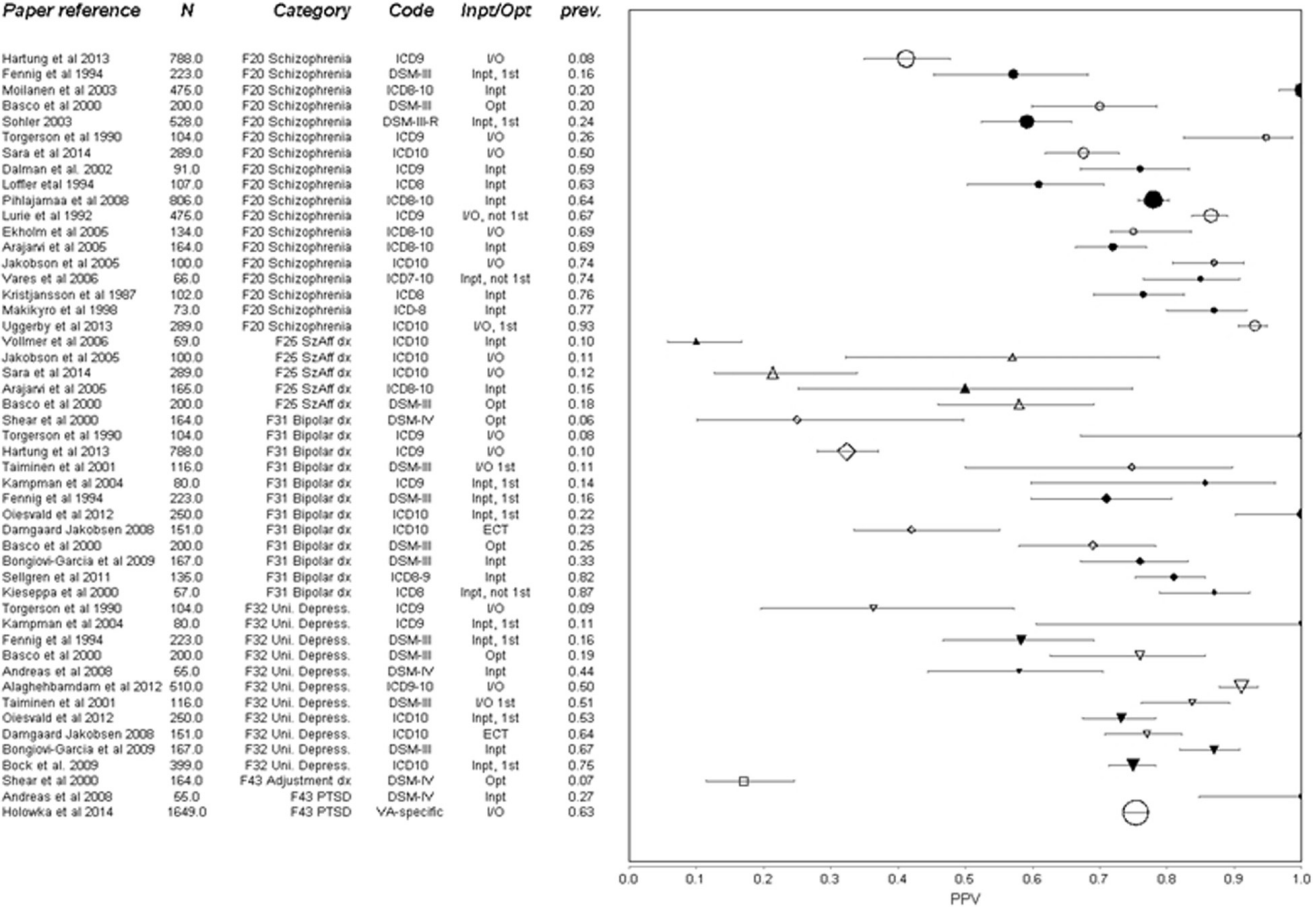
Forest Plot of positive predictive value (PPV) of administrative diagnoses for the respective research diagnosis at disorder level. Whiskers represent 95 % confidence intervals. Sorted by diagnosis (also indicated by *shape*), then prevalence in the sample *prev*. Diagnosis has been converted to nearest ICD-10 equivalent, with original code indicated. *Size* of marker represents square root of complete sample size N. Inpatient (Inpt), Outpatient (Opt) or mixed (I/O) origin of data indicated, with studies using only inpatient diagnoses also having *filled* marker. Studies using only people in their first episode of illness (1st) or selected on a history of ECT are indicated. Abbreviations: dx = disorder, SzAff = schizo-affective, Uni Depress = unipolar depression, PTSD = post-traumatic stress disorder

**Fig. 4:**
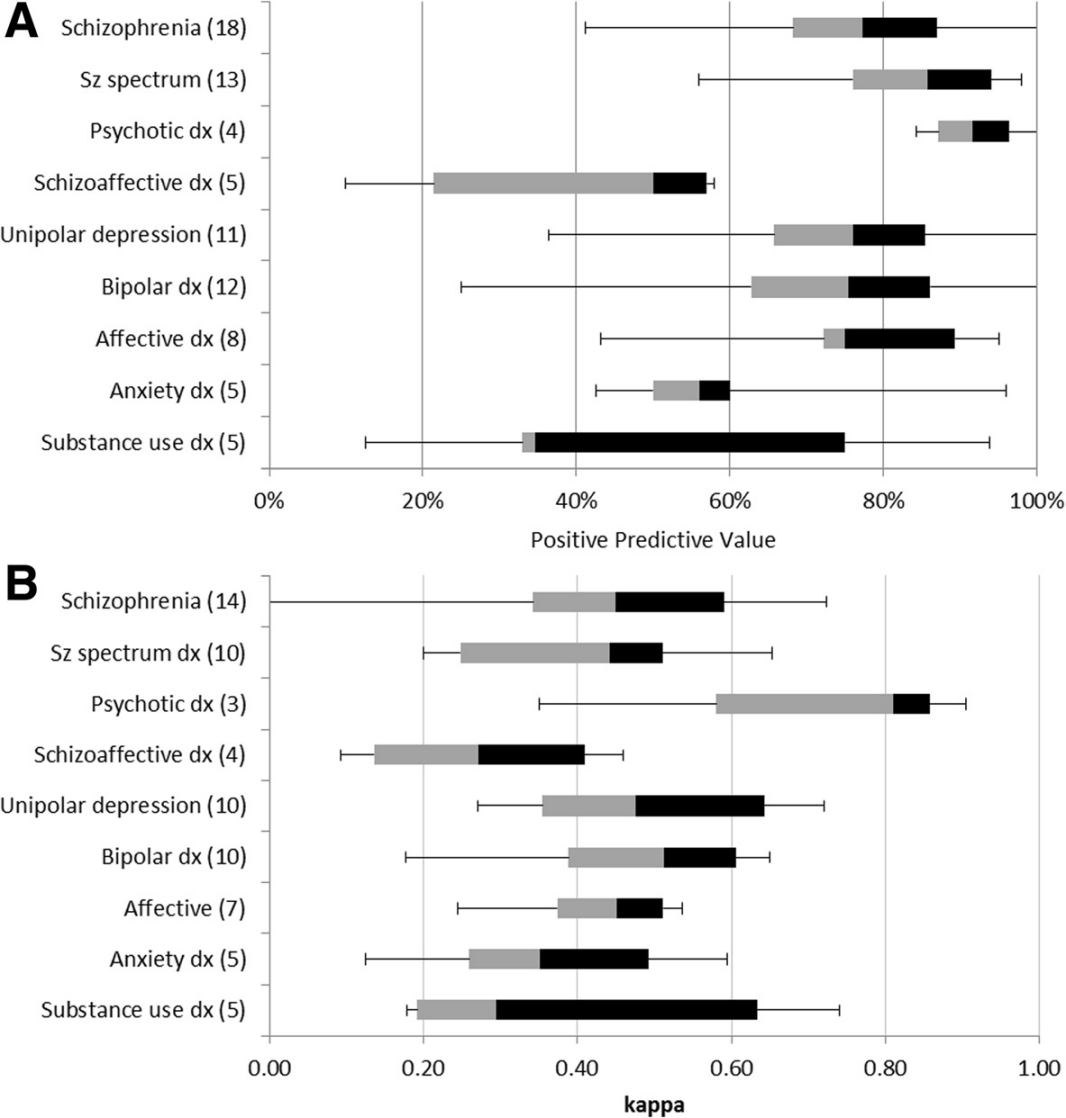
Box plots summarising **a** positive predictive values (PPV) and **b** Cohen’s kappa of diagnoses reported in four or more studies. Treating each result as a point, the median of PPVs is the transition line, the interquartile range is indicated by the *box* and the range of findings by the whiskers. Numbers of studies in parentheses

The Positive Predictive Value (PPV) for diagnosis for all studies is plotted by diagnostic group in the Forest Plots in Figs. 2 and 3, showing how the PPV varies by prevalence and diagnostic group amongst other variables. For those diagnostic categories with four or more results, the spread is also displayed in box plot Fig. 4a, where the range and quartile values of PPV results in the same diagnostic category can be seen. The spread of Cohen’s kappa is also shown for comparison in Fig. 4b.

The highest PPV was for the broad category of “psychotic” illness. Every study agreed that in a cohort with a diagnosis of psychotic illness recorded in secondary care, at least 80 % are likely to meet research criteria for this, and most suggested over 90 %. The diagnosis of schizophrenia shows a greater spread of PPV (40–100 %) than psychotic illness, but the majority of studies found the diagnosis at least 75 % predictive. “Schizophrenia spectrum” results lie in-between that of broad psychosis and narrow schizophrenia. Other diagnoses that have a median PPV around 75 % are affective disorders (with approximately the same spread as schizophrenia), unipolar depression and bipolar affective disorder (with a wider spread). Substance misuse disorders and anxiety disorders had a lower median PPV, while the diagnosis of schizoaffective disorder had a low PPV (<60 %) in all of the five studies that included it.

The variation of kappa within diagnostic category is very large, the range being lowest for affective disorders (0.3), and highest for affective disorders and highest for schizophrenia (0.7). But between diagnostic groups the variation is small compared with PPV. The median kappa for schizophrenia and schizophrenia spectrum disorders are both around 0.5, as are diagnoses of depression and bipolar disorder.

### Results by inpatient status

We divided studies into those done exclusively on inpatient data, and those that included both inpatients and outpatients. Since around half of the studies in the inpatient group looked only at patients in their first presentation, which might be expected to have lower accuracy, we subdivided into three groups: inpatient only, first presentation only, and mixed in/outpatient. To compare them, we considered only the most common diagnostic categories: the diagnosis-specific results for schizophrenia or schizophrenia spectrum (schizophrenia used in preference where both given); unipolar depression and bipolar, or affective disorder (individual diagnoses used in preference where given); and overall agreement. There were 25 diagnoses considered in the *mixed* group with median PPV 72 % (interquartile range 44–87), 13 results in the *inpatient* group with median PPV 77 % (IQR 76–85), and 20 results from *first* presentation with median PPV 75 % (IQR 71–93). Looking at kappa (median 0.50, 0.45 and 0.49 respectively) with Kruskall-Wallis pairwise comparison found no significant difference between inpatient and mixed, or between 1st and mixed presentations (*p* > 0.1).

## Discussion

### Review findings

We found thirty-nine studies on the accuracy of routinely collected data on diagnoses in mental health, of diverse design and quality. Error appeared to be significantly greater at the clinical/diagnostic stage than in the transfer to administrative data. The spread of results for both positive predictive value and agreement (kappa), even within one diagnosis, was very large. Never the less, it could be seen that for well-defined disorders such as schizophrenia, a moderately high predictive value could be expected (median ~75 %), especially when the diagnosis was made in the context of high prevalence. For diagnostic groups of anxiety and substance use disorders, and the diagnosis of schizoaffective disorder, there was on average less predictive value in the diagnosis in administrative data (although schizoaffective disorder as part of schizophrenia spectrum disorders is likely to be better). Kappa values ranged from negative to perfect, but median level for most disorders suggested moderate reliability. We did not find the expected advantage of an inpatient diagnosis, nor a disadvantage from first presentation in the most common diagnostic categories.

### Confidence in results

The papers reported in this review are mainly of moderate quality. Of the recommended items in the checklist [21], the papers between them scored 64, 67 and 47 % for the introduction, methods and reporting sections respectively. From a practical point of view, variations in study design hampered the integration of results and interpretation thereof. A concern for generalizability was that a sizable proportion of the papers were involved in validating a source of potential diagnoses in order to later use this source in their research, which could lead to publication bias – as there is little incentive to publish about a source that is rejected as invalid. Due to the differences in design of the larger vs smaller studies, a conventional analysis to look for publication bias is unlikely to be informative. The overall agreement levels of the studies compared was poorer than both predicted [65] and measured inter-rater reliability for psychiatric diagnoses, which suggests that some unfavourable results are being published.

We did not formally test for causes of heterogeneity between the studies, other than by diagnosis and inpatient status as above; but visualization of the Forest plots does not suggest a major contribution for age of study, diagnostic code used or location of study. It is possible that local clinical, administrative and other factors are major causes of heterogeneity in results that we found. Heterogeneity of study design is also likely to play a part.

A strength of our review was that we performed a comprehensive search, including forward bibliographic searching. We also decided to include a wide range of studies incorporating administrative and diagnostic error, past and present coding systems, and a large number of different psychiatric conditions. This allowed us to present the whole range of results that could be expected from a new source of diagnoses.

Weaknesses in this review are that the included studies were too heterogeneous to combine in order to adequately test for publication bias or changes over time, and the wide overlapping ranges for some of the categories that were compared leads to reduced confidence in the validity of our comparisons. We chose to concentrate on diagnoses made in secondary care, which is helpful for looking at severe mental illness, but not common disorders. We also limited our discussion to the ability to ‘rule in’ a disorder, rather than ruling out disorders, which is of use when creating control groups. Risk of bias was increased as the search and data extraction was carried out by just one co-author, and we did not register the review with any database of systematic reviews.

### Comparing with others

Byrne et al. [19], who carried out the only previous review of validation studies for psychiatric diagnosis using narrower inclusion criteria, found similar disparities in the reporting of different studies, and was not able to analyse further. Some of our other findings that can be compared with previous findings include that schizophrenia and schizophrenia spectrum are relatively reliable, since Chang et al. [66] in a review of the stability of psychosis diagnoses found that people with psychosis tend to shift towards schizophrenia over time. Our finding that the major source of error between clinician and administrative database was at the clinician stage was also found in Sytema et al., a study on three registries in the 1980s [67]. ‘Error’ for psychiatric diagnosis in our sample was between 0 and 90 %, which is probably higher than would be expected for medical diagnoses. The recent review of coding in NHS hospitals (excluding psychiatry) showed error in the primary diagnosis to be between 1 and 34 %, with a mean of 9 % [11].

### Psychiatric diagnosis in practice

Assigning a psychiatric diagnosis is not straight-forward. Although training and research concentrate on discrete categories, real cases are rarely simple to describe using these categories. Making a correct diagnosis depends on the depth and duration of observation over time and the range of information available [18]. The use of structured interviews improves reliability but is not widespread [68, 69]. There has also long been speculation that reliable categorical diagnosis in psychiatry is something of an illusion [70]. By this token, what we have termed as “diagnostic error” could be regarded as a demonstration of the weakness in the current diagnostic paradigm: the natural variation of practitioners forced to use artificial categories that do not reflect the issues of mental distress that they see.

If the “gold-standard” DSM/ICD diagnoses are not valid, then it would not be possible to calculate diagnostic validity, and our comparison would be measuring only reliability [71]. However, we have observed that inter-rater reliability for researchers is actually much higher than agreement between source and research diagnoses, meaning that error is occurring in clinical and administrative diagnosis as well as any problems there may be with the classifications per se.

While acknowledging that there is some merit in other ways of thinking about forms of normality and psychopathology, categorical diagnoses such as in DSM/ICD are still used to meaningfully communicate to other clinicians, allied health professionals and GPs, and have to be acceptable to all involved [15, 65]. Mental health diagnoses also frequently have legal consequences for individuals [72]. On a wider level, categorical diagnosis is used to inform managers and commissioners of the overall composition of a caseload. In research also, it is helpful that patients with specific sets of problems can be identified, for epidemiology, outcome studies, and recruitment into clinical trials. With the difficulties in assigning a specific diagnosis, and the diagnosis having to perform various legal, pecuniary and practical tasks, there have been reports of bias [73–76], and it should not be surprising that there is less reliability in routinely recorded diagnoses than those given by a disinterested party for research purposes.

## Conclusions

Our results suggest that administrative data is variable in its accuracy for diagnosis, and it may not be possible to generalise from one data source to another. Each data source may need to be validated individually, and this will enable the researcher to choose the outcomes most pertinent for their research needs. Following specific guidelines for validation studies would assist others to benefit [21]. The biggest source of error seems to be in the reliability of the clinical diagnostic process. One can be more confident in the diagnosis of psychotic disorders in general, and schizophrenia in particular, while great caution would have to be used in our view before concluding anything from administrative data about anxiety or substance disorders, or schizoaffective disorder. There may be issues about the meaningfulness of diagnostic classification in psychiatry that deserve further research, especially around the borders of these categories.

A register of patients is easier than ever to produce. Our finding, if replicated by others, that a register/administrative diagnosis may not be significantly less accurate than one recorded routinely in clinical notes (when compared to a reference diagnosis) may be of significance to researchers who can now access structured information in anonymised and pseudo-anonymised records through research databases [5, 77].

Despite our support for using administrative data in general, it should never be used unsupported to estimate incidence, prevalence, or disease burden in a population, as they are biased towards those who recognise a problem, seek help or become unmanageable in the community. The World Health Organization estimates that 35–50 % of people with mental disorders of high severity and disability have not seen a professional in the previous year [78]. Rather, we have identified that routine data from secondary care often has the power to identify likely cases of severe mental illness for further analysis. Those wishing to use administrative data for research purposes would do well to look for validity studies for their source data and their items of interest. Where there are gaps in the current evidence for commonly used sources, validation of diagnostic data should be attempted where possible.

## Abbreviations

DSM, diagnostic and statistical manual of mental disorders; HES, hospital episode statistics (England); ICD, WHO International Classification of Diseases; NPV, negative predictive value – probability condition absent given identified as negative; PPV, positive predictive value – probability condition present given identified as positive; PRISMA, preferred reporting items for systematic reviews and meta-analyses; r^2^, residual sum of squares, also referred to as coefficient of determination

## Additional files

Additional file 1: Figure S1. Search strategy (medline). (TIF 146 kb) Additional file 2: Figure S2. Search strategy (EMBASE). (TIF 570 kb) Additional file 3: Table S1. Quality assessment of papers meeting inclusion criteria, after Benchimol et al. [21]. (DOCX 58 kb) Additional file 4: Table S2. Excluded studies. (DOCX 75 kb) Additional file 5: Table S3. Included studies. (DOCX 59 kb)
