# Supplementary figures and images for: Can mental health diagnoses in administrative data be used for research? A systematic review of the accuracy of routinely collected diagnoses
Source: BMC Psychiatry. 2016 Jul 26;16:263. doi: 10.1186/s12888-016-0963-x (PMC4960739; doi:10.1186/s12888-016-0963-x)

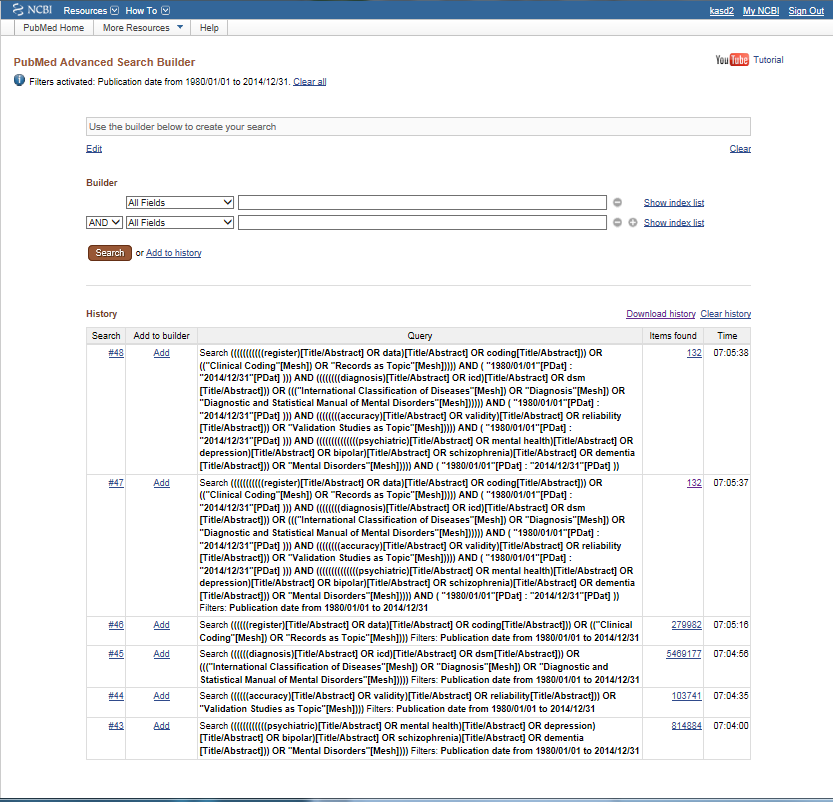

Supplement: Additional file 1: Figure S1. — Search strategy (medline). (TIF 146 kb) [file 12888_2016_963_MOESM1_ESM.tif]

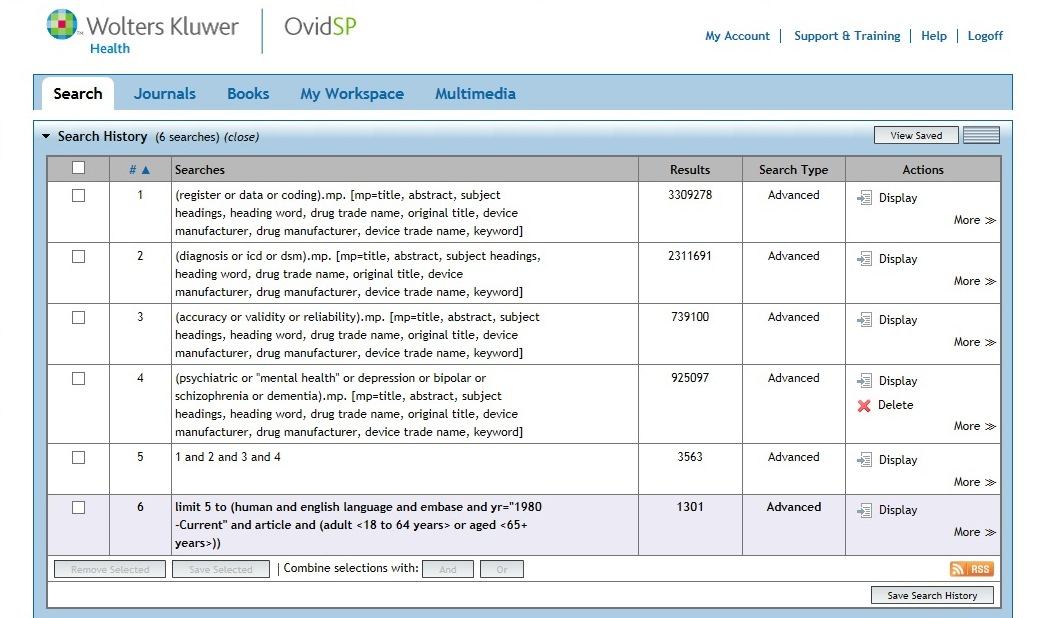

Supplement: Additional file 2: Figure S2. — Search strategy (EMBASE). (TIF 570 kb) [file 12888_2016_963_MOESM2_ESM.tif]
