# Supplementary material for: Can mental health diagnoses in administrative data be used for research? A systematic review of the accuracy of routinely collected diagnoses
Source: BMC Psychiatry. 2016 Jul 26;16:263. doi: 10.1186/s12888-016-0963-x (PMC4960739; doi:10.1186/s12888-016-0963-x)
Supplement: Additional file 3: Table S1. — Quality assessment of papers meeting inclusion criteria, after Benchimol et al. [21]. (DOCX 58 kb) [file 12888_2016_963_MOESM3_ESM.docx]

## Supplementary material Table s1: Quality assessment of papers meeting inclusion criteria

Criteria from [Benchimol et al. (2011)](#_ENREF_6) --

Intro: Identifies study as (1) assessing diagnostic accuracy (2) using administrative data (3) goal as identification or validation of disease.

Method: Describes: (1) participants; (2) selection (3) sampling (4) data collection (5) expertise of those reviewing, (6) statistical methods. Was there (7) blinding (8) split cohort (9) Kappa for experts.

Results and discussion: Reports (1) dates (2) numbers (3) distribution of severity (4) at least four estimates of diagnostic accuracy (5) confidence intervals. Includes (6) study flow diagram (7) cross-tabulation (8) comparison to prevalence (9) subgroup analysis (10) discussion of applicability

| Paper reference | Intro (3) | Method (9) | Results & Discussion (10) | **Total** |  |
| --- | --- | --- | --- | --- | --- |
| [Alaghehbandan et al. (2012)](#_ENREF_1) | 3 | 6.375 | 5 | **14.375** | moderate |
| [Andreas et al. (2009)](#_ENREF_2) | 2 | 5.625 | 5 | **12.625** | moderate |
| [Arajärvi et al. (2005)](#_ENREF_3) | 3 | 5.25 | 4 | **12.25** | moderate |
| [Balestrieri et al. (1997)](#_ENREF_4) | 1.5 | 6.75 | 2 | **10.25** | inadequate |
| [Basco et al. (2000)](#_ENREF_5) | 1.5 | 6 | 6 | **13.5** | poor |
| [Bock et al. (2009)](#_ENREF_7) | 3 | 5 | 6 | **14** | moderate |
| [Bongiovi-Garcia et al. (2009)](#_ENREF_8) | 2.5 | 6.5 | 3.5 | **12.5** | moderate |
| [Dalman et al. (2002)](#_ENREF_9) | 3 | 4.25 | 5 | **12.25** | moderate |
| [Damgaard Jakobsen et al. (2008)](#_ENREF_10) | 2.5 | 5.125 | 5 | **12.625** | moderate |
| [Ekholm et al. (2005)](#_ENREF_11) | 2 | 5.25 | 4 | **11.25** | moderate |
| [Fennig et al. (1994)](#_ENREF_12) | 2.5 | 7.5 | 5.5 | **15.5** | moderate |
| [Goodman et al. (1984)](#_ENREF_13) | 3 | 7.125 | 1.5 | **11.625** | inadequate |
| [Hartung et al. (2013)](#_ENREF_14) | 2.5 | 4 | 6.5 | **13** | moderate |
| [Holowka et al. (2014)](#_ENREF_15) | 3 | 7.25 | 6.5 | **16.75** | moderate |
| [Jakobsen et al. (2005)](#_ENREF_16) | 2 | 5 | 3 | **10** | poor |
| [Jörgensen et al. (2010)](#_ENREF_17) | 1 | 4.625 | 6 | **11.625** | inadequate |
| [Kampman et al. (2004)](#_ENREF_18) | 2.5 | 7.5 | 6.5 | **16.5** | moderate |
| [Kessing (1998)](#_ENREF_19) | 3 | 5 | 4 | **12** | moderate |
| [Kieseppa et al. (2000)](#_ENREF_20) | 3 | 6.375 | 5.5 | **14.875** | moderate |
| [Kristjansson et al. (1987)](#_ENREF_21) | 3 | 5.5 | 3.5 | **12** | moderate |
| [Lipton and Simon (1985)](#_ENREF_22) | 2.5 | 4.5 | 2.5 | **9.5** | inadequate |
| [Löffler et al. (1994)](#_ENREF_23) | 2 | 4.125 | 5 | **11.125** | moderate |
| [Lurie et al. (1992)](#_ENREF_24) | 3 | 6.25 | 3 | **12.25** | moderate |
| [Mäkikyrö et al. (1998)](#_ENREF_25) | 3 | 6.375 | 4.5 | **13.875** | moderate |
| [McConville and Walker (2000)](#_ENREF_26) | 3 | 7.5 | 5 | **15.5** | moderate |
| [Moilanen et al. (2003)](#_ENREF_27) | 3 | 7.375 | 8 | **18.375** | good |
| [Oiesvold et al. (2012)](#_ENREF_28) | 2.5 | 8.75 | 3 | **14.25** | moderate |
| [Perälä et al. (2007)](#_ENREF_29) | 1 | 6.375 | 7.5 | **14.875** | poor |
| [Pihlajamaa et al. (2008)](#_ENREF_30) | 3 | 4.5 | 5.5 | **13** | moderate |
| [Pulver et al. (1988)](#_ENREF_31) | 2 | 4.625 | 3 | **9.625** | moderate |
| [Quan et al. (2008)](#_ENREF_32) | 3 | 6.375 | 5 | **14.375** | moderate |
| [Rawson et al. (1997)](#_ENREF_33) | 3 | 5.75 | 4.5 | **13.25** | moderate |
| [Robinson and Tataryn (1997)](#_ENREF_34) | 3 | 5 | 2.5 | **10.5** | moderate |
| [Sara et al. (2014)](#_ENREF_35) | 2.5 | 5.75 | 6.5 | **14.75** | moderate |
| [Sellgren et al. (2011)](#_ENREF_36) | 3 | 6.25 | 4 | **13.25** | moderate |
| [Shear et al. (2000)](#_ENREF_37) | 1.5 | 5.625 | 5.5 | **12.625** | poor |
| [Sohler and Bromet (2003)](#_ENREF_38) | 2 | 6.375 | 6 | **14.375** | moderate |
| [Taiminen et al. (2001)](#_ENREF_39) | 2.5 | 7.5 | 5.5 | **15.5** | moderate |
| [Torgersen et al. (1990)](#_ENREF_40) | 2 | 6.25 | 4 | **12.25** | moderate |
| [Uggerby et al. (2013)](#_ENREF_41) | 3 | 5.125 | 3 | **11.125** | moderate |
| [Vares et al. (2006)](#_ENREF_42) | 2.5 | 7.75 | 2 | **12.25** | moderate |
| [Vollmer-Larsen et al. (2006)](#_ENREF_43) | 2 | 6 | 5 | **13** | moderate |
| [Walkup et al. (2000)](#_ENREF_44) | 2.5 | 3.875 | 3.5 | **9.875** | poor |

ALAGHEHBANDAN, R., MACDONALD, D., BARRETT, B., COLLINS, K. & CHEN, Y. 2012. Using Administrative Databases in the Surveillance of Depressive Disorders—Case Definitions. *Population Health Management,* 15**,** 372-380.

ANDREAS, S., THEISEN, P., MESTEL, R., KOCH, U. & SCHULZ, H. 2009. Validity of routine clinical DSM-IV diagnoses (Axis I/II) in inpatients with mental disorders. *Psychiatry Research,* 170**,** 252-255.

ARAJÄRVI, R., SUVISAARI, J., SUOKAS, J., SCHRECK, M., HAUKKA, J., HINTIKKA, J., PARTONEN, T. & LÖNNQVIST, J. 2005. Prevalence and diagnosis of schizophrenia based on register, case record and interview data in an isolated Finnish birth cohort born 1940–1969. *Social Psychiatry and Psychiatric Epidemiology,* 40**,** 808-816.

BALESTRIERI, M., RUCCI, P. & NICOLAOU, S. 1997. Gender-specific decline and seasonality of births in operationally defined schizophrenics in italy. *Schizophrenia Research,* 27**,** 73-81.

BASCO, M. R., BOSTIC, J. Q., DAVIES, D., RUSH, A. J., WITTE, B., HENDRICKSE, W. & BARNETT, V. 2000. Methods to improve diagnostic accuracy in a community mental health setting. *American Journal of Psychiatry,* 157**,** 1599-1605.

BENCHIMOL, E. I., MANUEL, D. G., TO, T., GRIFFITHS, A. M., RABENECK, L. & GUTTMANN, A. 2011. Development and use of reporting guidelines for assessing the quality of validation studies of health administrative data. *J Clin Epidemiol,* 64**,** 821-9.

BOCK, C., BUKH, J., VINBERG, M., GETHER, U. & KESSING, L. 2009. Validity of the diagnosis of a single depressive episode in a case register. *Clinical Practice and Epidemiology in Mental Health,* 5**,** 4.

BONGIOVI-GARCIA, M. E., MERVILLE, J., ALMEIDA, M. G., BURKE, A., ELLIS, S., STANLEY, B. H., POSNER, K., MANN, J. J. & OQUENDO, M. A. 2009. Comparison of clinical and research assessments of diagnosis, suicide attempt history and suicidal ideation in major depression. *Journal of Affective Disorders,* 115**,** 183-188.

DALMAN, C., BROMS, J., CULLBERG, J. & ALLEBECK, P. 2002. Young cases of schizophrenia identified in a national inpatient register. *Social Psychiatry and Psychiatric Epidemiology,* 37**,** 527-531.

DAMGAARD JAKOBSEN, K., HANSEN, T., DAM, H., BUNDGAARD LARSEN, E., GETHER, U. & WERGE, T. 2008. Reliability of clinical ICD-10 diagnoses among electroconvulsive therapy patients with chronic affective disorders. *The European Journal of Psychiatry,* 22**,** 161-172.

EKHOLM, B., EKHOLM, A., ADOLFSSON, R., VARES, M., ÖSBY, U., SEDVALL, G. C. & JÖNSSON, E. G. 2005. Evaluation of diagnostic procedures in Swedish patients with schizophrenia and related psychoses. *Nordic Journal of Psychiatry,* 59**,** 457-464.

FENNIG, S., CRAIG, T. J., TANENBERG-KARANT, M. & BROMET, E. J. 1994. Comparison of facility and research diagnoses in first-admission psychotic patients. *Am J Psychiatry,* 151**,** 1423-9.

GOODMAN, A. B., RAHAV, M., POPPER, M., GINATH, Y. & PEARL, E. 1984. The reliability of psychiatric diagnosis in Israel's Psychiatric Case Register. *Acta Psychiatrica Scandinavica,* 69**,** 391-397.

HARTUNG, D. M., MIDDLETON, L., MCFARLAND, B. H., HAXBY, D. G., MCDONAGH, M. S. & MCCONNELL, J. 2013. Use of administrative data to identify off-label use of second-generation antipsychotics in a medicaid population. *Psychiatric Services,* 64**,** 1236-1242.

HOLOWKA, D. W., MARX, B. P., GATES, M. A., LITMAN, H. J., RANGANATHAN, G., ROSEN, R. C. & KEANE, T. M. 2014. PTSD diagnostic validity in Veterans Affairs electronic records of Iraq and Afghanistan veterans. *Journal of Consulting and Clinical Psychology,* 82**,** 569-579.

JAKOBSEN, K. D., FREDERIKSEN, J. N., HANSEN, T., JANSSON, L. B., PARNAS, J. & WERGE, T. 2005. Reliability of clinical ICD-10 schizophrenia diagnoses. *Nordic Journal of Psychiatry,* 59**,** 209-212.

JÖRGENSEN, L., AHLBOM, A., ALLEBECK, P. & DALMAN, C. 2010. The Stockholm non-affective psychoses study (snaps): the importance of including out-patient data in incidence studies. *Acta Psychiatrica Scandinavica,* 121**,** 389-392.

KAMPMAN, O., KIVINIEMI, P., KOIVISTO, E., VÄÄNÄNEN, J., KILKKU, N., LEINONEN, E. & LEHTINEN, K. 2004. Patient characteristics and diagnostic discrepancy in first-episode psychosis. *Comprehensive Psychiatry,* 45**,** 213-218.

KESSING, L. V. 1998. Validity of diagnoses and other clinical register data in patients with affective disorder. *European Psychiatry,* 13**,** 392-398.

KIESEPPA, T., PARTONEN, T., KAPRIO, J. & LONNQVIST, J. 2000. Accuracy of register- and record-based bipolar I disorder diagnoses in Finland; a study of twins. *Acta Neuropsychiatrica,* 12**,** 106-109.

KRISTJANSSON, E., ALLEBECK, P. & WISTEDT, B. 1987. Validity of the diagnosis schizophrenia in a psychiatric inpatient register: A retrospective application of DSM-III criteria on ICD-8 diagnoses in Stockholm county. *Nordic Journal of Psychiatry,* 41**,** 229-234.

LIPTON, A. A. & SIMON, F. S. 1985. Psychiatric Diagnosis in a State Hospital: Manhattan State Revisited. *Psychiatric Services,* 36**,** 368-373.

LÖFFLER, W., HÄFNER, H., FÄTKENHEUER, B., MAURER, K., RIECHER-RÖSSLER, A., LÜTZHØFT, J., SKADHEDE, S., MUNK-JØRGENSEN, P. & STRÖMGREN, E. 1994. Validation of Danish case register diagnosis for schizophrenia. *Acta Psychiatrica Scandinavica,* 90**,** 196-203.

LURIE, N., POPKIN, M., DYSKEN, M., MOSCOVICE, I. & FINCH, M. 1992. Accuracy of Diagnoses of Schizophrenia in Medicaid Claims. *Psychiatric Services,* 43**,** 69-71.

MÄKIKYRÖ, T., ISOHANNI, M., MORING, J., HAKKO, H., HOVATTA, I. & LÖNNQVIST, J. 1998. Accuracy of register-based schizophrenia diagnoses in a genetic study. *European Psychiatry,* 13**,** 57-62.

MCCONVILLE, P. & WALKER, N. P. 2000. The reliability of case register diagnoses: a birth cohort analysis. *Social Psychiatry and Psychiatric Epidemiology,* 35**,** 121-127.

MOILANEN, K., VEIJOLA, J., LÄKSY, K., MÄKIKYRÖ, T., MIETTUNEN, J., KANTOJÄRVI, L., KOKKONEN, P., KARVONEN, J. T., HERVA, A., JOUKAMAA, M., JÄRVELIN, M.-R., MORING, J., JONES, P. B. & ISOHANNI, M. 2003. Reasons for the diagnostic discordance between clinicians and researchers in schizophrenia in the Northern Finland 1966 Birth Cohort. *Social Psychiatry and Psychiatric Epidemiology,* 38**,** 305-310.

OIESVOLD, T., NIVISON, M., HANSEN, V., SORGAARD, K., OSTENSEN, L. & SKRE, I. 2012. Classification of bipolar disorder in psychiatric hospital. a prospective cohort study. *BMC Psychiatry,* 12**,** 13.

PERÄLÄ, J., SUVISAARI, J., SAARNI, S. I. & ET AL. 2007. LIfetime prevalence of psychotic and bipolar i disorders in a general population. *Archives of General Psychiatry,* 64**,** 19-28.

PIHLAJAMAA, J., SUVISAARI, J., HENRIKSSON, M., HEILÄ, H., KARJALAINEN, E., KOSKELA, J., CANNON, M. & LÖNNQVIST, J. 2008. The validity of schizophrenia diagnosis in the Finnish Hospital Discharge Register: Findings from a 10-year birth cohort sample. *Nordic Journal of Psychiatry,* 62**,** 198-203.

PULVER, A. E., CARPENTER, W. T., ADLER, L. & MCGRATH, J. 1988. Accuracy of the diagnoses of affective disorders and schizophrenia in public hospitals. *American Journal of Psychiatry,* 145**,** 218-220.

QUAN, H., LI, B., DUNCAN SAUNDERS, L., PARSONS, G. A., NILSSON, C. I., ALIBHAI, A., GHALI, W. A. & FOR THE, I. I. 2008. Assessing Validity of ICD-9-CM and ICD-10 Administrative Data in Recording Clinical Conditions in a Unique Dually Coded Database. *Health Services Research,* 43**,** 1424-1441.

RAWSON, N. S., MALCOLM, E. & D'ARCY, C. 1997. Reliability of the recording of schizophrenia and depressive disorder in the Saskatchewan health care datafiles. *Soc Psychiatry Psychiatr Epidemiol,* 32**,** 191-9.

ROBINSON, J. R. & TATARYN, D. 1997. Reliability of the Manitoba Mental Health Management Information System for Research. *Canadian journal of psychiatry. Revue canadienne de psychiatrie,* 42**,** 744-749.

SARA, G., LUO, L., CARR, V., RAUDINO, A., GREEN, M., LAURENS, K., DEAN, K., COHEN, M., BURGESS, P. & MORGAN, V. 2014. Comparing algorithms for deriving psychosis diagnoses from longitudinal administrative clinical records. *Social Psychiatry and Psychiatric Epidemiology***,** 1-9.

SELLGREN, C., LANDÉN, M., LICHTENSTEIN, P., HULTMAN, C. M. & LÅNGSTRÖM, N. 2011. Validity of bipolar disorder hospital discharge diagnoses: file review and multiple register linkage in Sweden. *Acta Psychiatrica Scandinavica,* 124**,** 447-453.

SHEAR, M. K., GREENO, C., KANG, J., LUDEWIG, D., FRANK, E., SWARTZ, H. A. & HANEKAMP, M. 2000. Diagnosis of nonpsychotic patients in community clinics. *American Journal of Psychiatry,* 157**,** 581-587.

SOHLER, N. P. & BROMET, E. P. 2003. Does racial bias influence psychiatric diagnoses assigned at first hospitalization? *Social Psychiatry and Psychiatric Epidemiology,* 38**,** 463-472.

TAIMINEN, T., RANTA, K., KARLSSON, H., LAUERMA, H., LEINONEN, K.-M., WALLENIUS, E., KALJONEN, A. & SALOKANGAS, R. K. R. 2001. Comparison of clinical and best-estimate research DSM-IV diagnoses in a Finnish sample of first-admission psychosis and severe affective disorder. *Nordic Journal of Psychiatry,* 55**,** 107-111.

TORGERSEN, T., ROSSELAND, L. A. & MALT, U. F. 1990. Coding guidelines for ICD-9 section on mental disorders and reliability of chart clinical diagnoses. *Acta Psychiatrica Scandinavica,* 81**,** 62-67.

UGGERBY, P., ØSTERGAARD, S. D., RØGE, R., CORRELL, C. U. & NIELSEN, J. 2013. The validity of the schizophrenia diagnosis in the Danish Psychiatric Central Research Register is good. *Danish medical journal,* 60**,** A4578-A4578.

VARES, M., EKHOLM, A., SEDVALL, G. C., HALL, H. & JÖNSSON, E. G. 2006. Characterization of Patients with Schizophrenia and Related Psychoses: Evaluation of Different Diagnostic Procedures. *Psychopathology,* 39**,** 286-295.

VOLLMER-LARSEN, A., JACOBSEN, T. B., HEMMINGSEN, R. & PARNAS, J. 2006. Schizoaffective disorder-- the reliability of its clinical diagnostic use. *Acta Psychiatr Scand,* 113**,** 402-7.

WALKUP, J., BOYER, C. & KELLERMANN, S. 2000. Reliability of Medicaid Claims Files for Use in Psychiatric Diagnoses and Service Delivery. *Administration and Policy in Mental Health and Mental Health Services Research,* 27**,** 129-139.
