# Supplementary material for: Can mental health diagnoses in administrative data be used for research? A systematic review of the accuracy of routinely collected diagnoses
Source: BMC Psychiatry. 2016 Jul 26;16:263. doi: 10.1186/s12888-016-0963-x (PMC4960739; doi:10.1186/s12888-016-0963-x)
Supplement: Additional file 4: Table S2. — Excluded studies. (DOCX 75 kb) [file 12888_2016_963_MOESM4_ESM.docx]

## Supplementary material Table s2: Excluded Papers – primary reason given, although may not meet inclusion criteria in multiple domains.

| Reason | Paper |
| --- | --- |
| Atypical methodology | Peabody et al. (2004)  Rezvyy et al. (2005) |
| Comorbidity (diagnosis in context of other disease) | Cepoiu et al. (2007)  Cornelius et al. (2014)  Egan et al. (2003)  Fazel et al. (2009)  Jasser et al. (2007)  Marrie et al. (2013) |
| Diagnostic technique (testing a diagnostic technique) | Del-Ben et al. (2005)  Erdman et al. (1987)  Helzer et al. (1978)  Kirkby et al. (1998)  Miller et al. (2001)  Steiner et al. (1995) |
| Emergency setting | Rufino et al. (2005)  Strakowski et al. (1997)  Suominen et al. (1999)  Taggart et al. (2006) |
| Inadequate quality | Balestrieri et al. (1997)  Goodman et al. (1984)  Jager et al. (2012)  Jörgensen et al. (2010)  Lipton and Simon (1985) |
| Mood state | Perlis et al. (2012)  Rothschild et al. (2008) |
| No gold-standard comparison | Barrera et al. (2014)  Bulloch et al. (2011)  Cho et al. (2014)  Choi et al. (2012)  Folsom et al. (2006)  Frayne et al. (2010)  Füredi et al. (2003)  Gambassi et al. (1998)  Grann and Holmberg (1999)  Hansen et al. (2000)  Joling et al. (2011)  Kamat et al. (2008)  Kendell et al. (1993)  Kiejna et al. (2014)  Lützhøft et al. (1995)  McCarron et al. (2014)  McGregor et al. (2010)  Mojtabai (2013)  Munk-Jørgensen (1986)  Noyes et al. (2011)  Parker et al. (1985)  Sytema et al. (1989) |
| Not psychiatric diagnosis | Fisher et al. (1992)  Green and Wintfeld (1993)  Kashner and Michael (1998)  Lloyd and Rissing (1985)  MacIntyre et al. (1997)  Poikolainen (1983)  Solberg et al. (2006) |
| Not routine diagnosis | Davison et al. (2009)  Eguale et al. (2010)  Leckman et al. (1982)  Mantere et al. (2004)  McGlashan (1984)  Neighbors et al. (2003)  Sartorius et al. (1986) |
| Pre DSM-III | Schwartz et al. (1980) |
| Primary care | Spettell et al. (2003)  Trinh et al. (2011) |
| Sample already included | Corty et al. (1993)  Fiest et al. (2014)  Isohanni et al. (1997) |
| Sensitivity only | Sorvaniemi et al. (1998)  World Mental Health Survey Consortium (2004) |
| Specific sub-population | Reutfors et al. (2010) |
| Stability of diagnosis | Andersen et al. (2013)  Anglin and Malaspina (2008)  Bergman et al. (1999)  Chang et al. (2009)  Chen et al. (1996)  Dhossche and Ghani (1998)  Jakobsen et al. (2007)  Möller et al. (2011) |

ANDERSEN, S., RANDERS, A., JENSEN, C., BISGAARD, C. & STEINHAUSEN, H.-C. 2013. Preceding diagnoses to young adult bipolar disorder and schizophrenia in a nationwide study. *BMC Psychiatry,* 13**,** 343.

ANGLIN, D. M. & MALASPINA, D. 2008. Racial and ethnic effects on psychotic psychiatric diagnostic changes from admission to discharge: A retrospective chart review. *Journal of Clinical Psychiatry,* 69**,** 464-469.

BALESTRIERI, M., RUCCI, P. & NICOLAOU, S. 1997. Gender-specific decline and seasonality of births in operationally defined schizophrenics in italy. *Schizophrenia Research,* 27**,** 73-81.

BARRERA, T. L., MOTT, J. M., HUNDT, N. E., MIGNOGNA, J., YU, H. J., STANLEY, M. A. & CULLY, J. A. 2014. Diagnostic specificity and mental health service utilization among veterans with newly diagnosed anxiety disorders. *General Hospital Psychiatry,* 36**,** 192-198.

BERGMAN, B., BELFRAGE, H. & GRANN, M. 1999. Mentally disordered offenders in Sweden: forensic and general psychiatric diagnoses. *American Journal of Forensic Psychiatry,* 20**,** 27-38.

BULLOCH, A. G., CURRIE, S., GUYN, L., WILLIAMS, J. V., LAVORATO, D. H. & PATTEN, S. B. 2011. Estimates of the treated prevalence of bipolar disorders by mental health services in the general population: comparison of results from administrative and health survey data. *Chronic Diseases and Injuries in Canada,* 31**,** 129-134.

CEPOIU, M., MCCUSKER, J., COLE, M. G., SEWITCH, M. & CIAMPI, A. 2007. Recognition of depression in older medical inpatients. *J Gen Intern Med,* 22**,** 559-64.

CHANG, W. C., CHAN, S. S. M. & CHUNG, D. W. S. 2009. Diagnostic stability of functional psychosis: A systematic review. *Hong Kong Journal of Psychiatry,* 19**,** 30-41.

CHEN, Y. R., SWANN, A. C. & BURT, D. B. 1996. Stability of Diagnosis in schizophrenia. *American Journal of Psychiatry,* 153**,** 682-686.

CHO, K., GAGNON, D. R., DRIVER, J. A., ALTINCATAL, A., KOSIK, N., LANES, S. & LAWLER, E. V. 2014. Dementia Coding, Workup, and Treatment in the VA New England Healthcare System. *International Journal of Alzheimer&#x2019;s Disease,* 2014**,** 5.

CHOI, M. R., EUN, H. J., YOO, T. P., YUN, Y., WOOD, C., KASE, M., PARK, J. I. & YANG, J. C. 2012. The effects of sociodemographic factors on psychiatric diagnosis. *Psychiatry Investig,* 9**,** 199-208.

CORNELIUS, B., VAN DER KLINK, J. J. L., BROUWER, S. & GROOTHOFF, J. W. 2014. Under-recognition and under-treatment of DSM-IV classified mood and anxiety disorders among disability claimants. *Disability and Rehabilitation,* 36**,** 1161-1168.

CORTY, E., LEHMAN, A. F. & MYERS, C. P. 1993. Influence of psychoactive substance use on the reliability of psychiatric diagnosis. *J Consult Clin Psychol,* 61**,** 165-70.

DAVISON, T. E., MCCABE, M. P. & MELLOR, D. 2009. An examination of the "gold standard" diagnosis of major depression in aged-care settings. *Am J Geriatr Psychiatry,* 17**,** 359-67.

DEL-BEN, C. M., HALLAK, J. E. C., SPONHOLZ JR, A., MARQUES, J. M. D. A., LABATE, C. M., CONTEL, J. O. B. & ZUARDI, A. W. 2005. Accuracy of psychiatric diagnosis performed under indirect supervision. *Revista Brasileira de Psiquiatria,* 27**,** 58-62.

DHOSSCHE, D. & GHANI, S. 1998. Diagnostic Stability of Schizophrenia in Psychiatric Emergency Room Patients. *Annals of Clinical Psychiatry,* 10**,** 151-155.

EGAN, S., NATHAN, P. & LUMLEY, M. 2003. Diagnostic Concordance of ICD-10 Personality and Comorbid Disorders: A Comparison of Standard Clinical Assessment and Structured Interviews in a Clinical Setting. *Australian and New Zealand Journal of Psychiatry,* 37**,** 484-491.

EGUALE, T., WINSLADE, N., HANLEY, J., BUCKERIDGE, D. & TAMBLYN, R. 2010. Enhancing Pharmacosurveillance with Systematic Collection of Treatment Indication in Electronic Prescribing. *Drug Safety,* 33**,** 559-567.

ERDMAN, H. P., KLEIN, M. H., GREIST, J. H., BASS, S. M., BIRES, J. K. & MACHTINGER, P. E. 1987. A comparison of the Diagnostic Interview Schedule and clinical diagnosis. *Am J Psychiatry,* 144**,** 1477-80.

FAZEL, S., LÅNGSTRÖM, N., HJERN, A., GRANN, M. & LICHTENSTEIN, P. 2009. SChizophrenia, substance abuse, and violent crime. *JAMA,* 301**,** 2016-2023.

FIEST, K. M., JETTE, N., QUAN, H., ST GERMAINE-SMITH, C., METCALFE, A., PATTEN, S. B. & BECK, C. A. 2014. Systematic review and assessment of validated case definitions for depression in administrative data. *BMC Psychiatry,* 14**,** 289.

FISHER, E. S., WHALEY, F. S., KRUSHAT, W. M., MALENKA, D. J., FLEMING, C., BARON, J. A. & HSIA, D. C. 1992. The accuracy of Medicare's hospital claims data: progress has been made, but problems remain. *American Journal of Public Health,* 82**,** 243-248.

FOLSOM, D. P., LINDAMER, L., MONTROSS, L. P., HAWTHORNE, W., GOLSHAN, S., HOUGH, R., SHALE, J. & JESTE, D. V. 2006. Diagnostic variability for schizophrenia and major depression in a large public mental health care system dataset. *Psychiatry Research,* 144**,** 167-175.

FRAYNE, S. M., MILLER, D. R., SHARKANSKY, E. J., JACKSON, V. W., WANG, F., HALANYCH, J. H., BERLOWITZ, D. R., KADER, B., ROSEN, C. S. & KEANE, T. M. 2010. Using Administrative Data to Identify Mental Illness: What Approach Is Best? *American Journal of Medical Quality,* 25**,** 42-50.

FÜREDI, J., RÓZSA, S., ZÁMBORI, J. & SZÁDÓCZKY, E. 2003. The Role of Symptoms in the Recognition of Mental Health Disorders in Primary Care. *Psychosomatics,* 44**,** 402-406.

GAMBASSI, G., LANDI, F., PENG, L., BROSTRUP-JENSEN, C., CALORE, K., HIRIS, J., LIPSITZ, L., MOR, V. & BERNABEI, R. 1998. Validity of Diagnostic and Drug Data in Standardized Nursing Home Resident Assessments: Potential for Geriatric Pharmacoepidemiology. *Medical Care,* 36**,** 167-179.

GOODMAN, A. B., RAHAV, M., POPPER, M., GINATH, Y. & PEARL, E. 1984. The reliability of psychiatric diagnosis in Israel's Psychiatric Case Register. *Acta Psychiatrica Scandinavica,* 69**,** 391-397.

GRANN, M. & HOLMBERG, G. 1999. Follow-Up of Forensic Psychiatric Legislation and Clinical Practice in Sweden 1988 to 1995. *International Journal of Law and Psychiatry,* 22**,** 125-131.

GREEN, J. & WINTFELD, N. 1993. How Accurate are Hospital Discharge Data for Evaluating Effectiveness of Care? *Medical Care,* 31**,** 719-731.

HANSEN, S. S., MUNK-JØRGENSEN, P., GULDBÆK, B., SOLGÅRD, T., LAUSZUS, K. S., ALBRECHTSEN, N., BORG, L., EGANDER, A., FAURHOLDT, K., GILBERG, A., GOSDEN, N. P., LORENZEN, J., RICHELSEN, B., WEISCHER, K. & BERTELSEN, A. 2000. Psychoactive substance use diagnoses among psychiatric in-patients. *Acta Psychiatrica Scandinavica,* 102**,** 432-438.

HELZER, J., CLAYTON, P., PAMBAKIAN, R. & WOODRUFF, R., JR. 1978. Concurrent Diagnostic Validity of a Structured Psychiatric Interview. *Arch Gen Psychiatry,* 35.

ISOHANNI, M., MÄKIKYRÖ, T., MORING, J., RÄSANEN, P., HAKKO, H., PARTANEN, U., KOIRANEN, M. & JONES, P. 1997. A comparison of clinical and research DSM-III-R diagnoses of schizophrenia in a Finnish national birth cohort. *Social Psychiatry and Psychiatric Epidemiology,* 32**,** 303-308.

JAGER, M., BURGER, D., BECKER, T. & FRASCH, K. 2012. Diagnosis of adjustment disorder: reliability of its clinical use and long-term stability. *Psychopathology,* 45**,** 305-9.

JAKOBSEN, K. D., HANSEN, T. & WERGE, T. 2007. Diagnostic stability among chronic patients with functional psychoses: An epidemiological and clinical study. *BMC Psychiatry,* 7.

JASSER, S. A., GARVIN, J. H., WIEDEMER, N., ROCHE, D. & GALLAGHER, R. M. 2007. Information Technology in Mental Health Research: Impediments and Implications in One Chronic Pain Study Population. *Pain Medicine,* 8**,** S176-S181.

JOLING, K. J., VAN MARWIJK, H. W. J., PIEK, E., DER HORST, H. E. V., PENNINX, B. W., VERHAAK, P. & VAN HOUT, H. P. J. 2011. Do GPs' medical records demonstrate a good recognition of depression? A new perspective on case extraction. *Journal of Affective Disorders,* 133**,** 522-527.

JÖRGENSEN, L., AHLBOM, A., ALLEBECK, P. & DALMAN, C. 2010. The Stockholm non-affective psychoses study (snaps): the importance of including out-patient data in incidence studies. *Acta Psychiatrica Scandinavica,* 121**,** 389-392.

KAMAT, S. A., RAJAGOPALAN, K., PETHICK, N., WILLEY, V., BULLANO, M. & HASSAN, M. 2008. Prevalence and humanistic impact of potential misdiagnosis of bipolar disorder among patients with major depressive disorder in a commercially insured population. *J Manag Care Pharm,* 14**,** 631-42.

KASHNER, T. & MICHAEL, P. J. 1998. Agreement Between Administrative Files and Written Medical Records: A Case of the Department of Veterans Affairs. *Medical Care,* 36**,** 1324-1336.

KENDELL, R. E., MALCOLM, D. E. & ADAMS, W. 1993. The problem of detecting changes in the incidence of schizophrenia. *The British Journal of Psychiatry,* 162**,** 212-8.

KIEJNA, A., MISIAK, B., ZAGDANSKA, M., DRAPALA, J., PIOTROWSKI, P., SZCZESNIAK, D., CHLADZINSKA-KIEJNA, S., CIALKOWSKA-KUZMINSKA, M. & FRYDECKA, D. 2014. Money matters: does the reimbursement policy for second-generation antipsychotics influence the number of recorded schizophrenia patients and the burden of stigmatization? *Social Psychiatry and Psychiatric Epidemiology,* 49**,** 531-539.

KIRKBY, K. C., HAY, D. A., DANIELS, B. A., JONES, I. H. & MOWRY, B. J. 1998. Comparison between Register and Structured Interview Diagnoses of Schizophrenia: A Case for Longitudinal Diagnostic Profiles. *Australian and New Zealand Journal of Psychiatry,* 32**,** 410-414.

LECKMAN, J. F., SHOLOMSKAS, D., THOMPSON, D., BELANGER, A. & WEISSMAN, M. M. 1982. Best estimate of lifetime psychiatric diagnosis: A methodological study. *Archives of General Psychiatry,* 39**,** 879-883.

LIPTON, A. A. & SIMON, F. S. 1985. Psychiatric Diagnosis in a State Hospital: Manhattan State Revisited. *Psychiatric Services,* 36**,** 368-373.

LLOYD, S. S. & RISSING, J. 1985. Physician and coding errors in patient records. *JAMA,* 254**,** 1330-1336.

LÜTZHØFT, J. H., SKADHEDE, S., FÄTKENHEUER, B., HÄFNER, H., LÖFFLER, W., RIECHER-RÖSSLER, A. & MAURER, K. 1995. Symptom Assessment in Casenotes and the Clinical Diagnosis of Schizophrenia. *Psychopathology,* 28**,** 131-139.

MACINTYRE, C. R., ACKLAND, M. J., CHANDRARAJ, E. J. & PILLA, J. E. 1997. Accuracy of ICD–9–CM codes in hospital morbidity data, Victoria: implications for public health research. *Australian and New Zealand Journal of Public Health,* 21**,** 477-482.

MANTERE, O., SUOMINEN, K., LEPPÄMÄKI, S., VALTONEN, H., ARVILOMMI, P. & ISOMETSÄ, E. 2004. The clinical characteristics of DSM-IV bipolar I and II disorders: baseline findings from the Jorvi Bipolar Study (JoBS). *Bipolar Disorders,* 6**,** 395-405.

MARRIE, R. A., FISK, J., YU, B. N., LEUNG, S., ELLIOTT, L., CAETANO, P., WARREN, S., EVANS, C., WOLFSON, C., SVENSON, L., TREMLETT, H., BLANCHARD, J., PATTEN, S., EPIDEMIOLOGY, F. T. C. T. I. T. & SCLEROSIS, I. O. C. O. M. 2013. Mental comorbidity and multiple sclerosis: validating administrative data to support population-based surveillance. *BMC Neurology,* 13**,** 16.

MCCARRON, K. K., REINHARD, M. J., BLOESER, K. J., MAHAN, C. M. & KANG, H. K. 2014. PTSD Diagnoses Among Iraq and Afghanistan Veterans: Comparison of Administrative Data to Chart Review. *Journal of Traumatic Stress,* 27**,** 626-629.

MCGLASHAN, T. H. 1984. Testing four diagnostic systems for schizophrenia. *Archives of general psychiatry,* 41**,** 141-144.

MCGREGOR, J., BROOKS, C., CHALASANI, P., CHUKWUMA, J., HUTCHINGS, H., LYONS, R. & LLOYD, K. 2010. The Health Informatics Trial Enhancement Project (HITE): Using routinely collected primary care data to identify potential participants for a depression trial. *Trials,* 11**,** 39.

MILLER, P. R., DASHER, R., COLLINS, R., GRIFFITHS, P. & BROWN, F. 2001. Inpatient diagnostic assessments: 1. Accuracy of structured vs. unstructured interviews. *Psychiatry Research,* 105**,** 255-264.

MOJTABAI, R. 2013. Clinician-identified depression in community settings: concordance with structured-interview diagnoses. *Psychother Psychosom,* 82**,** 161-9.

MÖLLER, H. J., JÄGER, M., RIEDEL, M., OBERMEIER, M., STRAUSS, A. & BOTTLENDER, R. 2011. The Munich 15-year follow-up study (MUFUSSAD) on first-hospitalized patients with schizophrenic or affective disorders: Assessing courses, types and time stability of diagnostic classification. *European Psychiatry,* 26**,** 231-243.

MUNK-JØRGENSEN, P. 1986. Decreasing first-admission rates of schizophrenia among males in Denmark from 1970 to 1984. *Acta Psychiatrica Scandinavica,* 73**,** 645-650.

NEIGHBORS, H. W., TRIERWEILER, S. J., FORD, B. C. & MUROFF, J. R. 2003. Racial Differences in DSM Diagnosis Using a Semi-Structured Instrument: The Importance of Clinical Judgment in the Diagnosis of African Americans. *Journal of Health and Social Behavior,* 44**,** 237-256.

NOYES, K., LIU, H., LYNESS, J. M. & FRIEDMAN, B. 2011. Medicare Beneficiaries With Depression: Comparing Diagnoses in Claims Data With the Results of Screening. *Psychiatric Services,* 62**,** 1159-1166.

PARKER, G., O'DONNELL, M. & WALTER, S. 1985. Changes in the diagnoses of the functional psychoses associated with the introduction of lithium. *The British Journal of Psychiatry,* 146**,** 377-82.

PEABODY, J. W., LUCK, J., JAIN, S., BERTENTHAL, D. & GLASSMAN, P. 2004. Assessing the Accuracy of Administrative Data in Health Information Systems. *Medical Care,* 42**,** 1066-1072.

PERLIS, R. H., IOSIFESCU, D. V., CASTRO, V. M., MURPHY, S. N., GAINER, V. S., MINNIER, J., CAI, T., GORYACHEV, S., ZENG, Q., GALLAGHER, P. J., FAVA, M., WEILBURG, J. B., CHURCHILL, S. E., KOHANE, I. S. & SMOLLER, J. W. 2012. Using electronic medical records to enable large-scale studies in psychiatry: treatment resistant depression as a model. *Psychological Medicine,* 42**,** 41-50.

POIKOLAINEN, K. 1983. Accuracy of hospital discharge data: five alcohol-related diseases. *Drug and Alcohol Dependence,* 12**,** 315-322.

REUTFORS, J., BAHMANYAR, S., JÖNSSON, E. G., EKBOM, A., NORDSTRÖM, P., BRANDT, L. & ÖSBY, U. 2010. Diagnostic profile and suicide risk in schizophrenia spectrum disorder. *Schizophrenia Research,* 123**,** 251-256.

REZVYY, G., OIESVOLD, T., PARNIAKOV, A. & OLSTAD, R. 2005. A comparative study of diagnostic practice in psychiatry in Northern Norway and Northwest Russia. *Social Psychiatry and Psychiatric Epidemiology,* 40**,** 316-323.

ROTHSCHILD, A. J., WINER, J., FLINT, A. J., MULSANT, B. H., WHYTE, E. M., HEO, M., FRATONI, S., GABRIELE, M., KASAPINOVIC, S. & MEYERS, B. S. 2008. Missed diagnosis of psychotic depression at 4 academic medical centers. *Journal of Clinical Psychiatry,* 69**,** 1293-1296.

RUFINO, A. C. T. B. F., UCHIDA, R. R., VILELA, J. A. A., MARQUES, J. M. A., ZUARDI, A. W. & DEL-BEN, C. M. 2005. Stability of the diagnosis of first-episode psychosis made in an emergency setting. *General Hospital Psychiatry,* 27**,** 189-193.

SARTORIUS, N., JABLENSKY, A., KORTEN, A., ERNBERG, G., ANKER, M., COOPER, J. E. & DAY, R. 1986. Early manifestations and first-contact incidence of schizophrenia in different cultures: A preliminary report on the initial evaluation phase of the WHO Collaborative Study on Determinants of Outcome of Severe Mental Disorders. *Psychological Medicine,* 16**,** 909-928.

SCHWARTZ, A. H., PERLMAN, B. B., PARIS, M., SCHMIDT, K. & THORNTON, J. C. 1980. Psychiatric diagnoses as reported to Medicaid and as recorded in patient charts. *American Journal of Public Health,* 70**,** 406-408.

SOLBERG, L. I., ENGEBRETSON, K. I., SPERL-HILLEN, J. M., HROSCIKOSKI, M. C. & O'CONNOR, P. J. 2006. Are Claims Data Accurate Enough to Identify Patients for Performance Measures or Quality Improvement? The Case of Diabetes, Heart Disease, and Depression. *American Journal of Medical Quality,* 21**,** 238-245.

SORVANIEMI, M., HELENIUS, H., ALHO, A., KESTI, S. & SALOKANGAS, R. K. R. 1998. Improved Diagnostic Assessment of Major Depression in Psychiatric Outpatient Care in Finland. *Psychiatric Services,* 49**,** 384-386.

SPETTELL, C. M., WALL, T. C., ALLISON, J., CALHOUN, J., KOBYLINSKI, R., FARGASON, R. & KIEFE, C. I. 2003. Identifying Physician-Recognized Depression from Administrative Data: Consequences for Quality Measurement. *Health Services Research,* 38**,** 1081-1102.

STEINER, J. L., TEBES, J. K., SLEDGE, W. H. & WALKER, M. L. 1995. A comparison of the structured clinical interview for DSM-III-R and clinical diagnoses. *J Nerv Ment Dis,* 183**,** 365-9.

STRAKOWSKI, S. M., HAWKINS, J. M., KECK JR, P. E., MCELROY, S. L., WEST, S. A., BOURNE, M. L., SAX, K. W. & TUGRUL, K. C. 1997. The effects of race and information variance on disagreement between psychiatric emergency service and research diagnoses in first-episode psychosis. *Journal of Clinical Psychiatry,* 58**,** 457-463.

SUOMINEN, K., ISOMETSÄ, E., HENRIKSSON, M., SUOKAS, J., OSTAMO, A. & LÖNNQVIST, J. 1999. Consultation versus research diagnoses of mental disorders among suicide attempters. *Nordic Journal of Psychiatry,* 53**,** 253-256.

SYTEMA, S., GIEL, R., HORN, G. H. M. M. T., BALESTRIERI, M. & DAVIES, N. 1989. The reliability of diagnostic coding in psychiatric case registers. *Psychological Medicine,* 19**,** 999-1006.

TAGGART, C., O'GRADY, J., STEVENSON, M., HAND, E., MC CLELLAND, R. & KELLY, C. 2006. Accuracy of diagnosis at routine psychiatric assessment in patients presenting to an accident and emergency department. *General Hospital Psychiatry,* 28**,** 330-335.

TRINH, N.-H. T., YOUN, S. J., SOUSA, J., REGAN, S., BEDOYA, C. A., CHANG, T. E., FAVA, M. & YEUNG, A. 2011. Using electronic medical records to determine the diagnosis of clinical depression. *International Journal of Medical Informatics,* 80**,** 533-540.

WORLD MENTAL HEALTH SURVEY CONSORTIUM, W. H. O. 2004. Prevalence, severity, and unmet need for treatment of mental disorders in the world health organization world mental health surveys. *JAMA,* 291**,** 2581-2590.
