# Supplementary material for: Can mental health diagnoses in administrative data be used for research? A systematic review of the accuracy of routinely collected diagnoses
Source: BMC Psychiatry. 2016 Jul 26;16:263. doi: 10.1186/s12888-016-0963-x (PMC4960739; doi:10.1186/s12888-016-0963-x)
Supplement: Additional file 5: Table S3. — Included studies. (DOCX 59 kb) [file 12888_2016_963_MOESM5_ESM.docx]

## Supplementary material Table s3: Included papers

* Reference diagnosis could be: Chart (taken directly from clinical records) or Research – split into those that used “notes” review, and those who used “interview” to make diagnosis.

Abbreviations:

dx = disorder/disorders

Depression = episode of depression not in context of bipolar affective disorder, approximating to ICD-10 F32-33, including psychotic depression where psychotic patients are in cohort, elsewhere described as “unipolar depression”

HDR = hospital discharge register

NOS = “not otherwise specified”, a diagnostic term used by DSM and ICD used to describe an illness that fits in a category but does not filfill criteria for any specified disorder within that category

PCR = psychiatric case register

PTSD = post-traumatic stress disorder

sp. = spectrum disorder

SzAff = schizoaffective

| **Paper reference** | **Cohort** | **Size**  **N** | **Output categories** | **Source data** | **Code** | **Reference diagnosis method*** | **Notes** |
| --- | --- | --- | --- | --- | --- | --- | --- |
| Alaghehbandan et al. (2012) | Canada: One hospital admission or two visits to a psychiatrist in last two years with diagnosis major depression, dysthymia or depressive disorder NOS + controls. Mean age 51y. | 510 | Depression | HDR and Billing data | ICD9 ICD10 | Notes |  |
| Andreas et al. (2009) | Germany: Non-psychotic inpatients, mean age 31y. | 55 | Substance use dx  Affective dx  Depression  Anxiety dx  PTSD | Clinical | DSM-IV | Interview |  |
| Arajärvi et al. (2005) | Finland: Discharge diagnosis schizophrenia spectrum. Age 46-58y. | 164 | Schizophrenia sp.  Schizophrenia  SzAff dx | HDR | ICD8 ICD9 ICD10 | Notes +/- interview | Part of genetic study of small region |
| Basco et al. (2000) | USA: Outpatient volunteers. Age 18-76 (mean 38) | 200 | Schizophrenia  SzAff dx  Bipolar affective dx  Depression  Overall | Clinical | DSM-III | Notes and interview | Results on comorbidity in Theodore et al 2012 |
| Bock et al. (2009) | Denmark: FIRST contact with mental health services, diagnosed with depression and prescribed antidepressants. Age 18-70. | 399 | Depression | PCR | ICD10 | Interview | Part of study into treatment for depression |
| Bongiovi-Garcia et al. (2009) | USA: Volunteers. Discharge diagnosis of bipolar or unipolar depression. Age 18-72 (mean 39). | 201 | Bipolar affective dx  Depression | Clinical | DSM-III | Notes and Interview |  |
| Dalman et al. (2002) | Sweden: Discharge diagnosis schizophrenia. Age <25. | 100 | Schizoprenia sp.  Schizophrenia | HDR | ICD9 | Notes |  |
| Damgaard Jakobsen et al. (2008) | Denmark: Patients with a history of ECT. Ave 20y contact with mental health services. Mean age 53y. | 155 | Bipolar affective dx  Depression | PCR | ICD10 | Notes and self-report questionnaires | Chronic affective disorders |
| Ekholm et al. (2005) | Sweden: Diagnosis schizophrenia spectrum as in or outpatient (but see note). Age 23-74y (mean 42y). | 143 | Schizophrenia sp. Schizophrenia | HDR and PCR | ICD8 ICD9 ICD10 | Notes and Interview | Only 9 or 173 patients found on outpatient register were not HDR. |
| Fennig et al. (1994) | USA: FIRST admission, diagnosis psychosis. Age 15-60y. | 223 | Schizophrenia sp. Schizophrenia  Bipolar affective dx  Depression  Overall | Clinical | DSM-III | Notes and interview | Part of prevalence study |
| Hartung et al. (2013) | USA: Patients enrolled on Medicaid, taking antipsychotics in the community and attended outpatient psychiatric clinic at least twice. | 788 | Schizophrenia  Bipolar affective dx | Billing data | ICD9 | Chart +/- notes | Part of prescribing study. Also included treatment-resistant depression, but data not included in this review. |
| Holowka et al. (2014) | USA: Veterans of Iraq and Afghan wars with code for PTSD in Veterans Affairs (VA) psychiatric notes PLUS controls. Age 22-69y (mean 38y). | 1649 | PTSD | Admin records | VA-specific code | Interview | Part of project VALOR |
| Jakobsen et al. (2005) | Denmark: Volunteer cohort with in or outpatient diagnosis of psychotic illness. Age 17-66y. | 100 | Schizophrenia sp. Schizophrenia  SzAff dx  Overall | Clinical | ICD10 | Notes +/- interview | Members of Danish Psychiatric Biobank. |
| Kampman et al. (2004) | Finland: FIRST admission, diagnosis psychosis. Mean age 33y | 80 | Schizophrenia sp.  Bipolar affective dx  Depression  Overall | Clinical | ICD9 | Notes and Interview | Also mentions acute and transient psychosis, but insufficient information to be included in review. |
| Kessing (1998) | Denmark: FIRST admission, diagnosis manic-depressive psychosis. Mean age 52 at admission, 77 at reference diagnosis. | 100 | Affective dxs | PCR | ICD8 | Notes and interview | Mapping the ICD-8 concept of “manic-depressive psychosis” onto ICD-10 affective disorder |
| Kieseppa et al. (2000) | Finland: Diagnosis Bipolar affective dx type I or SzAff manic-type on more than one discharge diagnosis. Age 36-60y. | 42 | Bipolar affective dx | HDR | ICD8 | Notes and interview | Part of twin study. |
| Kristjansson et al. (1987) | Sweden: Discharge diagnosis schizophrenia. Age 10-79. | 102 | Schizophrenia | HDR | ICD8 | Notes |  |
| Löffler et al. (1994) | Denmark: Discharge diagnosis schizophrenia spectrum. Aged under 60y. | 107 | Schizophrenia | HDR | ICD8 | Notes |  |
| Lurie et al. (1992) | USA: Patients on Medicaid with diagnosis chronic mental illness from one inpatient or more than one outpatient episode | 475 | Schizophrenia | Billing data | ICD9 | Notes | Study on Schizophrenia, other pts included as control group |
| Mäkikyrö et al. (1998) | Finland: Discharge diagnosis of schizophrenia spectrum dx, and no sibling also has this. Aged 24-29y. | 73 | Schizophrenia sp.  Schizophrenia | HDR | DSM-III-R | Notes | Part of genetic study. |
| McConville and Walker (2000) | UK: Entry in register, derived from secondary care mental health. Age 75. | 412 | Organic dx  Substance use dx  Schizophrenia sp.  Affective dx  Anxiety dx  Personality dx  Mental retardation  Overall | PCR | ICD7 ICD8 ICD9 ICD10 | Notes | Birth-year research cohort. |
| Moilanen et al. (2003) | Finland: Any entry on psychiatric HDR. Age 31y. | 475 | Psychotic dx  Schizophrenia | HDR | ICD8 ICD9 ICD10 | Notes | Birth-year research cohort. |
| Oiesvold et al. (2012) | Finland: FIRST mental health admission, admitted for 3+ days. Age 18-65 (mean 40y) | 250 | Substance use dx  Schizophrenia sp.  Affective dx  Bipolar affective dx  Depression  Anxiety dx | Clinical | ICD10 | Notes and interview |  |
| Perälä et al. (2007) | Finland: Discharge diagnosis psychotic illness. Age 30-79y, and 80+y sampled 2:1. | 238 | Psychotic dxs | HDR | ICD8 ICD9 ICD10 | Interview | Part of prevalence study |
| Pihlajamaa et al. (2008) | Finland: Discharge diagnosis schizophrenia spectrum. Age 38-48y | 806 | Schizophrenia sp.  Schizophrenia | HDR | ICD8 ICD9 ICD10 | Notes | Also discusses contradictory entries (eg Schizophrenia and Bipolar I in separate reports). |
| Pulver et al. (1988) | USA: FIRST admission with psychotic features. White ethnicity. Age 16-65y. | 137 | Schizophrenia sp. | Clinical | DSM-III | Notes and interview |  |
| Quan et al. (2008) | Canada: The International Methodology Consortium for Coded Health Information study (IMECCHI). Inpatient (psychiatric or general ward), looking at primary and comorbid diagnoses. Age 18+ | 4008 | Substance dx  Depression  Psychotic dx | Admin records | ICD9 ICD10 | Chart | Depression the worst recorded, due to depression frequently being a comorbid diagnosis that was not coded. Further paper Fiest et al 2014. |
| Rawson et al. (1997) | Canada: Discharge diagnosis of Schizophrenic psychosis from major state hospitals. Age 13-96. | 131 | Schizophrenia | Admin records | ICD9 | Chart | Also looked at nonspecific depressive disorder, but results specific to ICD-8/9. |
| Robinson and Tataryn (1997) | Canada: Sample of those receiving secondary care mental health care, stratified by care received (inpatients oversampled) | 140 | Overall | Admin records | ICD9 | Chart | Looked at accuracy of demographic variables also |
| Sara et al. (2014) | Australia: Sub-sample of larger study. Screened positive for self-reported psychosis or randomly selected. Age 19-65 (mean 40). | 289 | Schizophrenia  SzAff dx  Affective dx  Psychotic dx | Admin records | ICD10 | Interview | Using the last or most often recorded diagnosis performed best where there were multiple records. |
| Sellgren et al. (2011) | Sweden: Two discharge diagnosis “manic-depressive psychosis” and none with schizophrenia. | 135 | Bipolar affective dx | HDR | ICD8 ICD9 | Notes | Accuracy could be further improved by disregarding “depressive type” relapses |
| Shear et al. (2000) | USA: Outpatients undergoing assessment who did not have psychotic symptoms. Aged 18-65y. | 114 | Substance use dx  Affective dx  Bipolar affective dx  Anxiety dx  Adjustment dx  Eating dx  Overall | Clinical | DSM-IV | Notes and interview | Discusses clinical implications of diagnostic error |
| Sohler and Bromet (2003) | USA: FIRST admission, prescribed antipsychotic or had documented psychotic features. White or Black ethnic group. Mean age around 30. | 528 | Schizophrenia  Affective dx | Clinical | DSM-III-R | Notes and interview | Suffolk County Mental Health Project. Did not find the expected racial bias. |
| Taiminen et al. (2001) | Finland: FIRST diagnosis psychotic illness (from admission or outpatient department, 85% inpatient). Age 16-64 (mean 35). | 116 | Schizophrenia sp.  Bipolar affective dx  Depression  Overall | Clinical | DSM-III | Notes and interview |  |
| Torgersen et al. (1990) | Finland: Patients in secondary care (in:outpatient 3:2). Age 18-86 (mean 40) | 104 | Psychotic dx | Clinical | ICD9 | Notes | Suggests abuse by clinicians of some diagnoses |
| Uggerby et al. (2013) | Denmark: FIRST diagnosis schizophrenia as in or out-patient | 291 | Schizophrenia | PCR | ICD10 | Notes | Reports admin and clinical error separately (13:7). |
| Vares et al. (2006) | Sweden: Current diagnosis schizophrenia spectrum (treatment period 4 months to 41y, mean 15.6y). Age 20-69y, mean 40. | 229 | Schizophrenia sp.  Schizophrenia | Clinical | ICD7 ICD8 ICD9 ICD10 | Notes and Interview | Used ‘hierarchical’ approach to multiple register diagnoses over time. Study looking re different methods of coming to research diagnosis. |
| Vollmer-Larsen et al. (2006) | Denmark: Discharge diagnosis SzAff dx. Age 24-81 (mean 48). | 59 | SzAff dx | HDR | ICD10 | Notes | Questions the validity of the clinical use of SzAff dx diagnosis. |
| Walkup et al. (2000) | USA: People with Medicaid claim for inpatient psychiatric treatment in New York State. Age over 18y. | 69 | Overall | Billing data | ICD9 | Chart |  |

ALAGHEHBANDAN, R., MACDONALD, D., BARRETT, B., COLLINS, K. & CHEN, Y. 2012. Using Administrative Databases in the Surveillance of Depressive Disorders—Case Definitions. *Population Health Management,* 15**,** 372-380.

ANDREAS, S., THEISEN, P., MESTEL, R., KOCH, U. & SCHULZ, H. 2009. Validity of routine clinical DSM-IV diagnoses (Axis I/II) in inpatients with mental disorders. *Psychiatry Research,* 170**,** 252-255.

ARAJÄRVI, R., SUVISAARI, J., SUOKAS, J., SCHRECK, M., HAUKKA, J., HINTIKKA, J., PARTONEN, T. & LÖNNQVIST, J. 2005. Prevalence and diagnosis of schizophrenia based on register, case record and interview data in an isolated Finnish birth cohort born 1940–1969. *Social Psychiatry and Psychiatric Epidemiology,* 40**,** 808-816.

BASCO, M. R., BOSTIC, J. Q., DAVIES, D., RUSH, A. J., WITTE, B., HENDRICKSE, W. & BARNETT, V. 2000. Methods to improve diagnostic accuracy in a community mental health setting. *American Journal of Psychiatry,* 157**,** 1599-1605.

BOCK, C., BUKH, J., VINBERG, M., GETHER, U. & KESSING, L. 2009. Validity of the diagnosis of a single depressive episode in a case register. *Clinical Practice and Epidemiology in Mental Health,* 5**,** 4.

BONGIOVI-GARCIA, M. E., MERVILLE, J., ALMEIDA, M. G., BURKE, A., ELLIS, S., STANLEY, B. H., POSNER, K., MANN, J. J. & OQUENDO, M. A. 2009. Comparison of clinical and research assessments of diagnosis, suicide attempt history and suicidal ideation in major depression. *Journal of Affective Disorders,* 115**,** 183-188.

DALMAN, C., BROMS, J., CULLBERG, J. & ALLEBECK, P. 2002. Young cases of schizophrenia identified in a national inpatient register. *Social Psychiatry and Psychiatric Epidemiology,* 37**,** 527-531.

DAMGAARD JAKOBSEN, K., HANSEN, T., DAM, H., BUNDGAARD LARSEN, E., GETHER, U. & WERGE, T. 2008. Reliability of clinical ICD-10 diagnoses among electroconvulsive therapy patients with chronic affective disorders. *The European Journal of Psychiatry,* 22**,** 161-172.

EKHOLM, B., EKHOLM, A., ADOLFSSON, R., VARES, M., ÖSBY, U., SEDVALL, G. C. & JÖNSSON, E. G. 2005. Evaluation of diagnostic procedures in Swedish patients with schizophrenia and related psychoses. *Nordic Journal of Psychiatry,* 59**,** 457-464.

FENNIG, S., CRAIG, T. J., TANENBERG-KARANT, M. & BROMET, E. J. 1994. Comparison of facility and research diagnoses in first-admission psychotic patients. *Am J Psychiatry,* 151**,** 1423-9.

HARTUNG, D. M., MIDDLETON, L., MCFARLAND, B. H., HAXBY, D. G., MCDONAGH, M. S. & MCCONNELL, J. 2013. Use of administrative data to identify off-label use of second-generation antipsychotics in a medicaid population. *Psychiatric Services,* 64**,** 1236-1242.

HOLOWKA, D. W., MARX, B. P., GATES, M. A., LITMAN, H. J., RANGANATHAN, G., ROSEN, R. C. & KEANE, T. M. 2014. PTSD diagnostic validity in Veterans Affairs electronic records of Iraq and Afghanistan veterans. *Journal of Consulting and Clinical Psychology,* 82**,** 569-579.

JAKOBSEN, K. D., FREDERIKSEN, J. N., HANSEN, T., JANSSON, L. B., PARNAS, J. & WERGE, T. 2005. Reliability of clinical ICD-10 schizophrenia diagnoses. *Nordic Journal of Psychiatry,* 59**,** 209-212.

KAMPMAN, O., KIVINIEMI, P., KOIVISTO, E., VÄÄNÄNEN, J., KILKKU, N., LEINONEN, E. & LEHTINEN, K. 2004. Patient characteristics and diagnostic discrepancy in first-episode psychosis. *Comprehensive Psychiatry,* 45**,** 213-218.

KESSING, L. V. 1998. Validity of diagnoses and other clinical register data in patients with affective disorder. *European Psychiatry,* 13**,** 392-398.

KIESEPPA, T., PARTONEN, T., KAPRIO, J. & LONNQVIST, J. 2000. Accuracy of register- and record-based bipolar I disorder diagnoses in Finland; a study of twins. *Acta Neuropsychiatrica,* 12**,** 106-109.

KRISTJANSSON, E., ALLEBECK, P. & WISTEDT, B. 1987. Validity of the diagnosis schizophrenia in a psychiatric inpatient register: A retrospective application of DSM-III criteria on ICD-8 diagnoses in Stockholm county. *Nordic Journal of Psychiatry,* 41**,** 229-234.

LÖFFLER, W., HÄFNER, H., FÄTKENHEUER, B., MAURER, K., RIECHER-RÖSSLER, A., LÜTZHØFT, J., SKADHEDE, S., MUNK-JØRGENSEN, P. & STRÖMGREN, E. 1994. Validation of Danish case register diagnosis for schizophrenia. *Acta Psychiatrica Scandinavica,* 90**,** 196-203.

LURIE, N., POPKIN, M., DYSKEN, M., MOSCOVICE, I. & FINCH, M. 1992. Accuracy of Diagnoses of Schizophrenia in Medicaid Claims. *Psychiatric Services,* 43**,** 69-71.

MÄKIKYRÖ, T., ISOHANNI, M., MORING, J., HAKKO, H., HOVATTA, I. & LÖNNQVIST, J. 1998. Accuracy of register-based schizophrenia diagnoses in a genetic study. *European Psychiatry,* 13**,** 57-62.

MCCONVILLE, P. & WALKER, N. P. 2000. The reliability of case register diagnoses: a birth cohort analysis. *Social Psychiatry and Psychiatric Epidemiology,* 35**,** 121-127.

MOILANEN, K., VEIJOLA, J., LÄKSY, K., MÄKIKYRÖ, T., MIETTUNEN, J., KANTOJÄRVI, L., KOKKONEN, P., KARVONEN, J. T., HERVA, A., JOUKAMAA, M., JÄRVELIN, M.-R., MORING, J., JONES, P. B. & ISOHANNI, M. 2003. Reasons for the diagnostic discordance between clinicians and researchers in schizophrenia in the Northern Finland 1966 Birth Cohort. *Social Psychiatry and Psychiatric Epidemiology,* 38**,** 305-310.

OIESVOLD, T., NIVISON, M., HANSEN, V., SORGAARD, K., OSTENSEN, L. & SKRE, I. 2012. Classification of bipolar disorder in psychiatric hospital. a prospective cohort study. *BMC Psychiatry,* 12**,** 13.

PERÄLÄ, J., SUVISAARI, J., SAARNI, S. I. & ET AL. 2007. LIfetime prevalence of psychotic and bipolar i disorders in a general population. *Archives of General Psychiatry,* 64**,** 19-28.

PIHLAJAMAA, J., SUVISAARI, J., HENRIKSSON, M., HEILÄ, H., KARJALAINEN, E., KOSKELA, J., CANNON, M. & LÖNNQVIST, J. 2008. The validity of schizophrenia diagnosis in the Finnish Hospital Discharge Register: Findings from a 10-year birth cohort sample. *Nordic Journal of Psychiatry,* 62**,** 198-203.

PULVER, A. E., CARPENTER, W. T., ADLER, L. & MCGRATH, J. 1988. Accuracy of the diagnoses of affective disorders and schizophrenia in public hospitals. *American Journal of Psychiatry,* 145**,** 218-220.

QUAN, H., LI, B., DUNCAN SAUNDERS, L., PARSONS, G. A., NILSSON, C. I., ALIBHAI, A., GHALI, W. A. & FOR THE, I. I. 2008. Assessing Validity of ICD-9-CM and ICD-10 Administrative Data in Recording Clinical Conditions in a Unique Dually Coded Database. *Health Services Research,* 43**,** 1424-1441.

RAWSON, N. S., MALCOLM, E. & D'ARCY, C. 1997. Reliability of the recording of schizophrenia and depressive disorder in the Saskatchewan health care datafiles. *Soc Psychiatry Psychiatr Epidemiol,* 32**,** 191-9.

ROBINSON, J. R. & TATARYN, D. 1997. Reliability of the Manitoba Mental Health Management Information System for Research. *Canadian journal of psychiatry. Revue canadienne de psychiatrie,* 42**,** 744-749.

SARA, G., LUO, L., CARR, V., RAUDINO, A., GREEN, M., LAURENS, K., DEAN, K., COHEN, M., BURGESS, P. & MORGAN, V. 2014. Comparing algorithms for deriving psychosis diagnoses from longitudinal administrative clinical records. *Social Psychiatry and Psychiatric Epidemiology***,** 1-9.

SELLGREN, C., LANDÉN, M., LICHTENSTEIN, P., HULTMAN, C. M. & LÅNGSTRÖM, N. 2011. Validity of bipolar disorder hospital discharge diagnoses: file review and multiple register linkage in Sweden. *Acta Psychiatrica Scandinavica,* 124**,** 447-453.

SHEAR, M. K., GREENO, C., KANG, J., LUDEWIG, D., FRANK, E., SWARTZ, H. A. & HANEKAMP, M. 2000. Diagnosis of nonpsychotic patients in community clinics. *American Journal of Psychiatry,* 157**,** 581-587.

SOHLER, N. P. & BROMET, E. P. 2003. Does racial bias influence psychiatric diagnoses assigned at first hospitalization? *Social Psychiatry and Psychiatric Epidemiology,* 38**,** 463-472.

TAIMINEN, T., RANTA, K., KARLSSON, H., LAUERMA, H., LEINONEN, K.-M., WALLENIUS, E., KALJONEN, A. & SALOKANGAS, R. K. R. 2001. Comparison of clinical and best-estimate research DSM-IV diagnoses in a Finnish sample of first-admission psychosis and severe affective disorder. *Nordic Journal of Psychiatry,* 55**,** 107-111.

TORGERSEN, T., ROSSELAND, L. A. & MALT, U. F. 1990. Coding guidelines for ICD-9 section on mental disorders and reliability of chart clinical diagnoses. *Acta Psychiatrica Scandinavica,* 81**,** 62-67.

UGGERBY, P., ØSTERGAARD, S. D., RØGE, R., CORRELL, C. U. & NIELSEN, J. 2013. The validity of the schizophrenia diagnosis in the Danish Psychiatric Central Research Register is good. *Danish medical journal,* 60**,** A4578-A4578.

VARES, M., EKHOLM, A., SEDVALL, G. C., HALL, H. & JÖNSSON, E. G. 2006. Characterization of Patients with Schizophrenia and Related Psychoses: Evaluation of Different Diagnostic Procedures. *Psychopathology,* 39**,** 286-295.

VOLLMER-LARSEN, A., JACOBSEN, T. B., HEMMINGSEN, R. & PARNAS, J. 2006. Schizoaffective disorder-- the reliability of its clinical diagnostic use. *Acta Psychiatr Scand,* 113**,** 402-7.

WALKUP, J., BOYER, C. & KELLERMANN, S. 2000. Reliability of Medicaid Claims Files for Use in Psychiatric Diagnoses and Service Delivery. *Administration and Policy in Mental Health and Mental Health Services Research,* 27**,** 129-139.
